# Management of CAR-T cell therapy in patients with multiple myeloma: a systematic review and expert consensus in Australia

**DOI:** 10.3389/fonc.2024.1535869

**Published:** 2025-01-21

**Authors:** P. Joy Ho, Hang Quach, M. Hasib Sidiqi, Cindy H. Lee, Jason Butler, Andrew Spencer, Kenneth Micklethwaite, Jingya Li, Elissa Cusson, Robert Bagnall, Simon J. Harrison

**Affiliations:** ^1^ Institute of Haematology, Royal Prince Alfred Hospital and University of Sydney, Sydney, NSW, Australia; ^2^ Department of Hematology, St. Vincent’s Hospital Melbourne and University of Melbourne, Melbourne, VIC, Australia; ^3^ Curtin Medical School, Curtin University, Perth, WA, Australia; ^4^ Haematology Department, Fiona Stanley Hospital, Murdoch, WA, Australia; ^5^ Department of Clinical Haematology, Royal Adelaide Hospital and University of Adelaide, Adelaide, SA, Australia; ^6^ Department of Haematology and Bone Marrow Transplant, Royal Brisbane and Women’s Hospital, Herston, QLD, Australia; ^7^ Department of Clinical Haematology, Alfred Hospital, Melbourne, VIC, Australia; ^8^ Blood Transplant and Cell Therapies Program, Department of Haematology, Westmead, NSW, Australia; ^9^ NSW Health Pathology Blood Transplant and Cell Therapies Laboratory – Institute Of Clinical Pathology And Medical Research (ICPMR) Westmead, Sydney, NSW, Australia Hospital, Sydney, NSW, Australia; ^10^ Westmead Institute for Medical Research, Sydney, NSW, Australia; ^11^ Sydney Medical School, The University of Sydney, Sydney, NSW, Australia; ^12^ Health Economics and Market Access, Amaris Consulting, Shanghai, China; ^13^ Health Economics and Market Access, Amaris Consulting, Barcelona, Spain; ^14^ Clinical Haematology, Peter MacCallum Cancer Centre and Royal Melbourne Hospital, Sir Peter MacCallum Department of Oncology, University of Melbourne, Melbourne, VIC, Australia

**Keywords:** chimeric antigen receptor immunotherapy, CAR-T cell therapy, systematic review, Delphi study, multiple myeloma, consensus development, Australia

## Abstract

**Background:**

Regulatory bodies have recently approved chimeric antigen receptor (CAR)-T cell therapies for patients with multiple myeloma (MM), but the treatment process involves complex decision making. To support the introduction of these therapies, we aimed to establish consensus expert opinion on best practices of all aspects of the management of patients with MM undergoing CAR-T cell therapy in Australia.

**Methods:**

We conducted a modified RAND/UCLA two-round Delphi panel informed by a systematic literature review (SLR). The SLR included evidence from clinical practice guidelines, interventional trials, and observational studies for CAR-T cell therapy for patients with MM, to synthesize methodological aspects of CAR-T cell therapy related to patient management. The Delphi panel comprised eight hematologists from across Australia, each with significant experience directly treating patients using CAR-T therapy or referring patients for CAR-T cell therapy. Panelists completed the surveys electronically, and attended a virtual meeting held before the second-round questionnaire to discuss the first-round questionnaire responses. Consensus was defined *a priori* as at least 70% agreement on survey questions.

**Results:**

The SLR identified 22 interventional or observational studies and 5 clinical practice guidelines reporting on selection and management of patients with MM treated with CAR-T cell therapy from various global regions. The Delphi panel reached consensus on practices related to patient referral, screening, selection, prioritization, treatments requiring wash-out, bridging therapy, lymphodepletion, infusion, and post-infusion monitoring and management. Most consensus results aligned with consistently recommended practices within guidelines included in the SLR. Consensus was not reached for statements related to specific screening practices and post-treatment monitoring, suggesting differing opinions on the specific best practices to implement.

**Conclusion:**

Our Delphi panel established expert consensus on key considerations for patient selection, administrative processes, and aftercare for patients with MM in Australia undergoing CAR-T therapy. This will guide the development of clinical practice guidelines which are relevant and feasible to Australian health systems.

## 1 Introduction

Multiple myeloma (MM) is a cancer of plasma cells and, as of 2022, was the second most common hematologic malignancy in Australia following lymphoma (1). Treatment recommendations for MM include chemotherapy, immunomodulatory drugs (IMiDs), proteasome inhibitors (PIs), corticosteroids, monoclonal antibodies, selective inhibitors of nuclear export (SINEs), and autologous stem cell transplantation (ASCT) for patients who meet age and fitness eligibility criteria (2, 3). However, agents such as bispecific antibodies and antibody-drug conjugates are not yet reimbursed by the Pharmaceutical Benefits Scheme (PBS) in Australia (3). Clinical practice guidelines recommend that Chimeric antigen receptor (CAR)-T cell therapies be considered for patients who have relapsed or are refractory to four prior lines of therapy (3, 4).

In June 2023, ciltacabtagene autoleucel (cilta-cel) received approval from the Therapeutic Goods Administration (TGA) for treatment of triple-class exposed patients with MM relapsing or refractory after three or more lines of therapy (5). With the approval by the US Food and Drug Administration (FDA) of idecabtagene vicleucel (ide-cel) in April 2024 for triple class exposed patients with relapsed or refractory multiple myeloma (RRMM) after at least two prior lines of treatment (6), and the approval of cilta-cel for patients with RRMM after at least one prior line of therapy (including a PI and an IMiD who are refractory to lenalidomide (6)), it is possible that these therapies could be available in earlier lines in Australia in the future.

In Australia, CAR-T cell therapy for RRMM typically involves a referral process coordinated among hematologists and specialized treatment centers (7). CAR-T cell therapy involves a complex sequence of critical steps, including patient evaluation and selection, leukapheresis and CAR-T cell production, lymphodepletion, infusion, monitoring for side effects such as cytokine release syndrome (CRS) and neurotoxic events such as immune effector cell-associated neurotoxicity syndrome (ICANS), and evaluation of treatment response and disease relapse (7–10). There is currently no Australian guideline or expert consensus on the clinical use of CAR-T cell therapy in patients with MM. To address this, we conducted a systematic literature review (SLR) of real-world evidence and clinical practice guidelines to identify optimal approaches to management of CAR-T cell therapy in patients with MM, based on which a Delphi panel was convened to seek consensus opinion on the most appropriate approaches for Australia.

## 2 Methods

### 2.1 Systematic literature review

EMBASE, MEDLINE, and MEDLINE In-Process were searched from inception to March 28, 2023 (**Supplementary Tables S1**, **S2**). Additional searches of clinical trial registries, conference proceedings, patient association websites, and Google Scholar were also conducted from January 1, 2020 to April 28, 2023. The population, intervention, comparator, outcomes, and study type (PICOS) framework was used to define prespecified study inclusion criteria (**Supplementary Table S3**) (11). The study selection process was documented in accordance with the preferred reporting items for systematic reviews and meta-analyses (PRISMA) statement 2020 (12). Full details of the SLR methodology can be found in **Supplementary Data Sheet 3**.

### 2.2 Delphi panel

We conducted a Delphi panel using a modified RAND/UCLA method with two rounds of survey and one intervening virtual meeting (13, 14). This method combines high-quality evidence with the collective judgment of experts to develop a statement regarding the appropriateness of a procedure or intervention. Eight hematologists representing five Australian states who had clinical experience using CAR-T therapy (treating patients with CAR-T cell therapy and/or referring patients for CAR-T therapy) were recruited as panelists. Panelists represented all Australian states and territories except the Northern Territory. The expert panelists electronically completed two rounds of questionnaires. Their responses were kept anonymous from the other panelists.

The questionnaire for the Delphi panel (see **Supplementary Data Sheets 1, 2**) comprised 10 questions exploring panelists’ opinions on the optimal practices related to patient referral, screening and selection, prioritization, treatment, and post-CAR-T management. Consensus was defined *a priori* as conformity by 70% of panelist responses, as used in De Meyer et al. (2019) (15). Five of the ten questions also assessed the panelists’ opinions on importance and practicality for the statements or practices with which consensus was reached. In the first round a five-point scale was applied and in the second round a three-point scale was used.

A virtual meeting was held after the first-round questionnaire. Questionnaire results were presented to the panelists and the statements that did not reach consensus were discussed. Panelists also provided suggestions on modifications of the questions and statements for the second-round questionnaire.

Panelists completed the second-round questionnaire with consideration of the outcomes from the first questionnaire and the virtual meeting. Panelists provided responses for statements that did not reach consensus in the first round as well as statements that needed clarification of importance and practicality, and new questions based on panelists’ input. Panelists indicated their opinion on the proposed cut-off values, timeframes, and strategies discussed during the first round. Cut-off threshold values were proposed to address patient- and disease-related factors relevant to referral and eligibility. Timeframes were suggested for steps preceding CAR-T cell infusion and for bridging therapy duration. Strategies to guide post-CAR-T monitoring were also discussed.

## 3 Results

### 3.1 Systematic literature review

#### 3.1.1 Identified studies and guidelines

**Figure 1:**
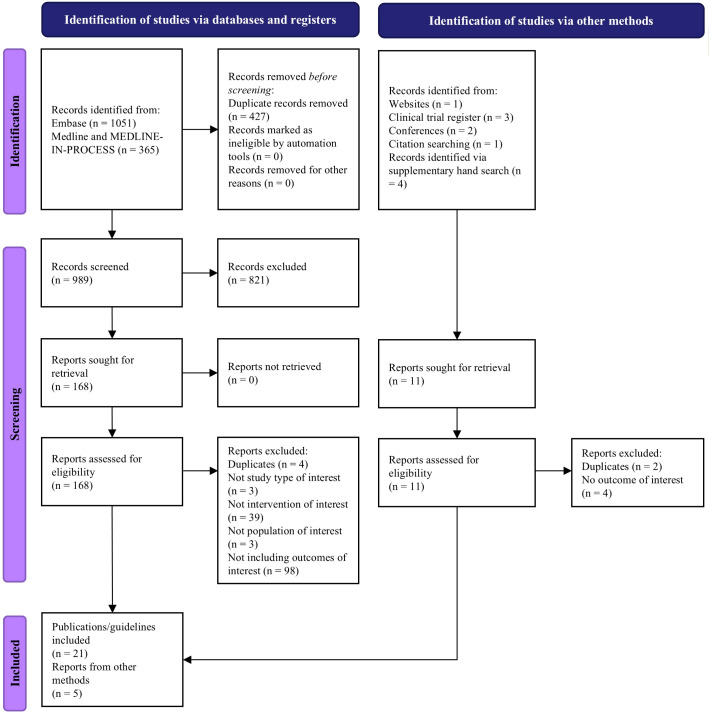
The study selection process based on PRISMA 2020 statement (12).

Of the 989 records screened, we included 26 reports in the SLR: 21 publications and 5 clinical practice guidelines (**Figure 1**). Publications had different scopes but mainly reported data from real-world settings or clinical trials (**Supplementary Table S4**). More publications reported data on ide-cel (n=9) compared to cilta-cel (n=5). The guidelines identified in our review were issued from the US, Brazil, Europe, China, and one international guideline from the American Society for Transplantation and Cellular Therapy (ASTCT), which included statements from experts from North America, Europe, Asia, and Australia (10, 16–19). All the guidelines were published between 2020 and 2023. The International Myeloma Working Group also published guidelines in 2024, but these were not available within the timeframe of this SLR (20).

#### 3.1.2 Patient referral, screening and selection, and prioritization

##### 3.1.2.1 Recommendations from current guidelines

None of the identified clinical practice guidelines discussed the referral process. Selection of patients with MM for CAR-T cell therapy requires evaluation of cardiac, respiratory, and renal function, an Eastern Cooperative Oncology Group (ECOG) performance score <2, and the absence of infection (10, 16–19). Guidelines stipulate that patients with active infections cannot initiate CAR-T cell therapy (16, 18). In relation to the previous lines of therapy, only the ASTCT guidelines offered a recommendation, stipulating that a minimum of four prior lines of treatment is required to be eligible for CAR-T therapy (17). A general neurological assessment and standard screening tests (routine blood counts, total bilirubin levels, and creatinine) are the main baseline evaluations that are recommended, along with serological testing for infections prior to leukapheresis, with test results available at the time of T-cell collection and shipment (16, 18, 19).

##### 3.1.2.2 Evidence from trials or real-world studies

None of the 21 included publications of clinical trials or real-world studies discussed the CAR-T cell therapy referral process. We identified five trials (three single-arm trials and two randomized clinical trials) (21–25) and two real-world studies (26, 27) that reported CAR-T cell therapy eligibility criteria for patients with MM. Common inclusion criteria were age, disease status, and diagnosis. For inclusion in clinical trials of CAR-T cell therapy for RRMM, patients were 18 years or older, had received 1 to 3 prior lines of treatment for cilta-cel or 2 to 4 for ide-cel, and had an ECOG performance score of 0 or 1 (21–25). Common exclusion criteria were comorbidities such as active hepatitis or cardiovascular diseases, and prior use of B-cell maturation antigen (BCMA)-targeting therapy (21–25). Although 75–90% of real-world patients in the US would not meet the inclusion criteria for clinical trials, the efficacy and toxicity outcomes from real-world evidence studies and trials were comparable (27).

Only one publication discussed prioritization. Most CAR-T cell therapy centers in the US (14/17) prioritized patients based on access to alternative therapy, with the most important ethical consideration being to maximize the benefit of CAR-T cell therapy by prioritizing patients most likely to survive until leukapheresis, dosing, and/or clinical response (28).

#### 3.1.3 Treatment management for CAR-T cell therapy in patients with MM

##### 3.1.3.1 Recommendations from current guidelines

Prior to leukapheresis, patients should be evaluated for full blood counts, organ function, performance status, and infections (10, 16–19). All guidelines recommended washout of prior treatment before apheresis, though recommendations differed regarding the duration of the washout periods. For example, if chemotherapy was administered, a washout period of at least 3–4 weeks was recommended in Europe (18), whereas 2 weeks were deemed sufficient in China (16). The type of bridging therapy also varied between guidelines. Guidelines from Brazil recommended steroids, PIs, alkylating agents, and monoclonal antibodies as bridging therapies (19), whereas guidelines from Europe recommended chemotherapy, radiotherapy, and novel agents (18).

Four guidelines recommended a combination of fludarabine and cyclophosphamide for lymphodepletion before CAR-T cell infusion (10, 16, 18, 19). Guidelines from China were the only ones to provide recommendations on timing of infusion, indicating that the infusion should start one to two days after lymphodepletion and not exceed seven days (16).

##### 3.1.3.2 Practices from trials or real-world studies

None of the studies described washout for treatments prior to CAR-T cell infusion. Fifteen studies discussed pre-CAR-T cell therapy management and infusion strategies. The choice of bridging therapies for disease control varied considerably, with a partial response rate of less than 33% and no complete responses to bridging reported (22–25, 27, 29–31). For lymphodepletion, standard regimens were 300 mg/m^2^/day cyclophosphamide and 30 mg/m^2^/day fludarabine, over 3 days and administered intravenously 5 to 7 days before CAR-T infusion, with potential need for dose modifications in patients with renal insufficiency (21, 22, 24, 25, 30, 32–34). The target infusion dose was 0.75×10^6^ cells/kg (range, 0.5–1.0×10^6^ cells/kg) for cilta-cel and 150–450×10^6^ cells for ide-cel (21, 23, 25, 32–34).

#### 3.1.4 Post-treatment management

##### 3.1.4.1 Recommendations from current guidelines

Monitoring strategies and management of toxicities for patients after CAR-T cell infusion were detailed in guidelines from the US, Brazil, Europe, and China. Recommendations on monitoring for cytopenias and toxicities, particularly CRS and neurological events including ICANS (10, 16, 18, 19), with hospitalization for 14 days post infusion was suggested in 2 guidelines (16, 18). Longer term follow-up (up to 15 years) for late onset toxicities (including prolonged or late cytopenias) was also recommended in Brazilian guidelines. During long-term monitoring, patients should be screened for dysplasia if they have cytopenias (19). European guidelines recommended monitoring CAR-T persistence through peripheral blood flow cytometry or molecular methods as clinically indicated (18).

##### 3.1.4.2 Practices from trials or real-world studies

Fifteen studies reported on post-infusion management, with neurotoxic/neurologic, infectious, and paraneoplastic complications being the most frequently monitored complications in patients with MM after CAR-T cell therapy. The monitoring strategies across studies followed similar trends, although the frequencies and durations varied (21, 24, 25). Short-term monitoring included daily assessments for the first 7 to 28 days and weekly assessments for the first 1 to 3 months. Long-term monitoring involved monthly assessments for 6 to 24 months after infusion, after which the frequency decreased to once every 3 to 12 months until disease progression (21, 24, 25).

The median incidence rate of neurotoxicity, including ICANS, was 15.7% across studies, ranging from 4.2% in China to 24% in the US (21, 23–25, 27, 30, 31, 33–36). Most MM patients receiving CAR-T cell infusion had hematologic events, and a median of 84% (range, 20–98%) had CRS (21, 23–25, 27, 30, 31, 34, 36).

### 3.2 Delphi panel

**Figure 2:**
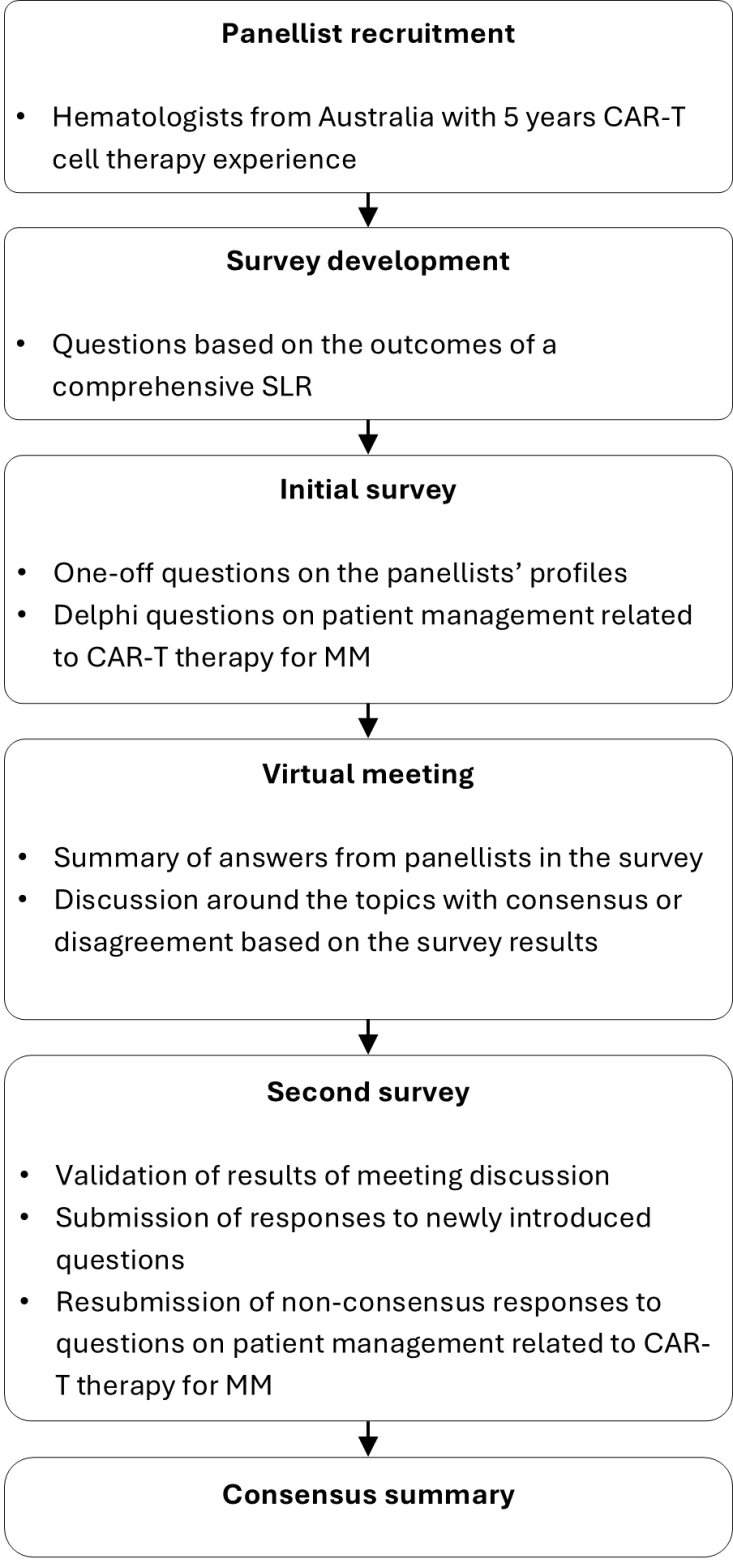
Flowchart of the modified Delphi panel.

Of the eight recruited panelists, seven completed the first Delphi panel questionnaire held from November 10, 2023, to February 14, 2024 (**Figure 2**). All eight panelists attended the subsequent virtual meeting on March 13, 2024. All eight panelists completed the second Delphi panel questionnaire conducted from March 27 to May 31, 2024.

#### 3.2.1 Patient referral, selection and prioritization

##### 3.2.1.1 Patient referral and selection

**Figure 3:**
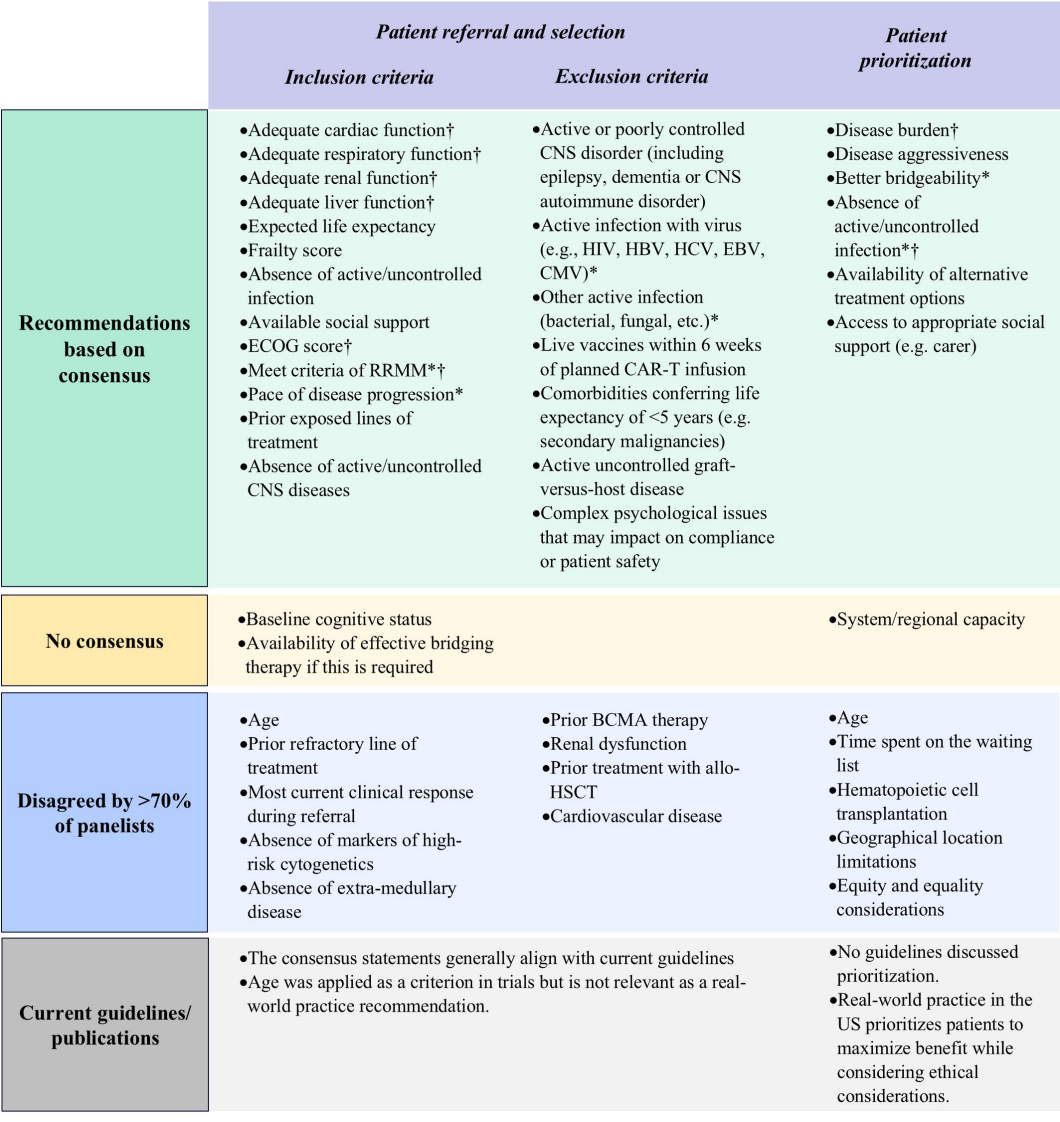
Results of Delphi survey on proposed factors for patient eligibility evaluation and prioritization. BCMA, B-cell maturation antigen; CAR-T, chimeric antigen receptor T-cell; CMV, cytomegalovirus; CNS, central nervous system; EBV, Epstein-Barr virus; ECOG, Eastern Cooperative Oncology Group; HBV, hepatitis B virus; HCV, hepatitis C virus; HIV, human immunodeficiency virus; RRMM, refractory-relapsing multiple myeloma. *Important factors as per panelists’ consensus of agreement; †Practical factors as per panelists’ consensus of agreement.

Delphi panelists agreed that key considerations for patient eligibility for referral should include cardiac, respiratory, renal, and liver function, absence of active and uncontrolled infection or CNS diseases, expected life expectancy, frailty score, social support, ECOG score, confirmation of relapse/refractory status, and pace of disease progression (**Figure 3**; **Supplementary Table S5**). Panelists also highlighted that earlier referrals are generally beneficial for the patient.

Panelists debated the criterion regarding infection and suggested that patients with active infection who are undergoing treatment be eligible for referral, agreeing that CAR-T therapy should only be initiated following resolution of the infection. The type of infection—whether chronic or requiring long-term treatment— would also influence the referral decision. Therefore, ‘absence of uncontrolled infection’ as a criterion for referral to CAR-T therapy was preferred over exclusion of patients with any infection at the referral stage.

Access to adequate caregiver support during treatment is another factor that should be considered prior to treatment initiation, but should not be a deciding factor for referral, as each center should try to ascertain whether external support can be made available.

Regarding factors that could exclude patients from receiving CAR-T therapy, panelists agreed that the presence of complex psychological issues, active or poorly controlled CNS disorder, active and uncontrolled viral (including HIV, HBV, HCV, EBV, CMV, or others), bacterial, or fungal infection, if they have received a dose of a live vaccine within the previous six weeks, comorbidities conferring a life expectancy of less than 5 years, and active and uncontrolled graft-versus-host disease (GvHD) should be considered (**Figure 3**; **Supplementary Table S6**). As latent HIV infection is not contraindicated for manufacturing of commercial and trial CAR-T products, patients with latent HIV infections can proceed to CAR-T cell therapy if they are receiving adequate anti-viral treatment. Renal impairment and cardiovascular disease should be effectively managed before initiating CAR-T therapy. However, these comorbidities should not be considered as strict exclusion criteria.

##### 3.2.1.2 Prioritization of selected patients

Panelists agreed that critical factors for patient prioritization should include disease burden, disease aggressiveness, bridgeability (the suitability of the patient to receive bridging therapy and likely outcome), absence of active and uncontrolled infection, availability of alternative treatment options, and access to appropriate social support including caregivers (**Figure 3**; **Supplementary Table S7**).

Panelists did not consider time spent on the waiting list, the Hematopoietic Cell Transplantation-Comorbidity Index (HCT-CI) score, age, and geographical limitations relevant for prioritization. The panelists emphasized that the timing of treatment should be considered on a case-by-case basis, with priority given to patients who are refractory or exhibit rapid progressive disease kinetics. Regarding geographical limitations, the panelists noted that patients can be cross-referred and should not be considered a factor for prioritization (**Figure 3**; **Supplementary Table S7**).

#### 3.2.2 Screening practices

Panelists agreed on the necessary screening practices at different stages before infusion. For patients who have been referred to CAR-T treatment centers, screening should include full blood counts, biochemistry, respiratory and cardiac function assessments, baseline neurocognitive tests, and a pregnancy test, if appropriate. Fewer than 30% of the panelists agreed on the use of whole-body MRI, CT, or PET-CT for screening. However, as one panelist noted, if the funding policy for these imaging procedures changes, then the recommendations surrounding their use could be reconsidered. Panelists also provided opinions on the required tests, thresholds, and timepoints of assessment, although they did not always reach consensus (**Table 1**; **Supplementary Table S8**).

**Table 1:** Cut-off values and suggested assessment timepoints that reached consensus of agreement.

Factor	Suggested approach
Adequate cardiac function	Evaluate before leukapheresis using LVEF ≥40% as cut-off value
Adequate respiratory function	Evaluate at the time of screening (no consensus on measurement and cut-off value)
Adequate renal function	Creatinine clearance ≥30ml/min as cut-off value (no consensus on timepoint)
Adequate liver function	Measure ALT, AST and total bilirubin (no consensus on cut-off value and timepoint)
Expected life expectancy	Evaluate before leukapheresis (no consensus on cut-off value)
Absence of active, uncontrolled infection	Detection of HIV, HBV, HCV, CMV, EBV, syphilis, and bacterial infections before leukapheresis and at lymphodepletion
ECOG score	Evaluate before lymphodepletion using ECOG <2 as cut-off value
Prior exposed lines of treatment	Align with reimbursement criteria/MSAC wording
Full Blood count	Hemoglobin (specific cut-off value not needed) and lymphocytes (no consensus on cut-off value and timepoint) should be evaluated
Full biochemistry	Corrected serum calcium should be evaluated (no consensus cut-off value)
Pregnancy test	Confirm as negative (no consensus on timepoint)

ALT, alanine aminotransferase; AST, aspartate aminotransferase; CMV, cytomegalovirus; EBV, Epstein-Barr virus; ECOG, Eastern Cooperative Oncology Group; HBV, hepatitis B virus; HCV, hepatitis C virus; HIV, human immunodeficiency virus; LVEF, left ventricular ejection fraction; MSAC, Medical Services Advisory Committee.

In addition, panelists agreed on the measurements for assessment of renal function, liver function, full biochemistry, and verifying number of prior lines of treatment. However, consensus on respective cut-off values and/or time points for these measurements were not reached (**Table 1**; **Supplementary Table S8**).

#### 3.2.3 Treatment management

##### 3.2.3.1 Wash-out prior to leukapheresis

**Figure 4:**
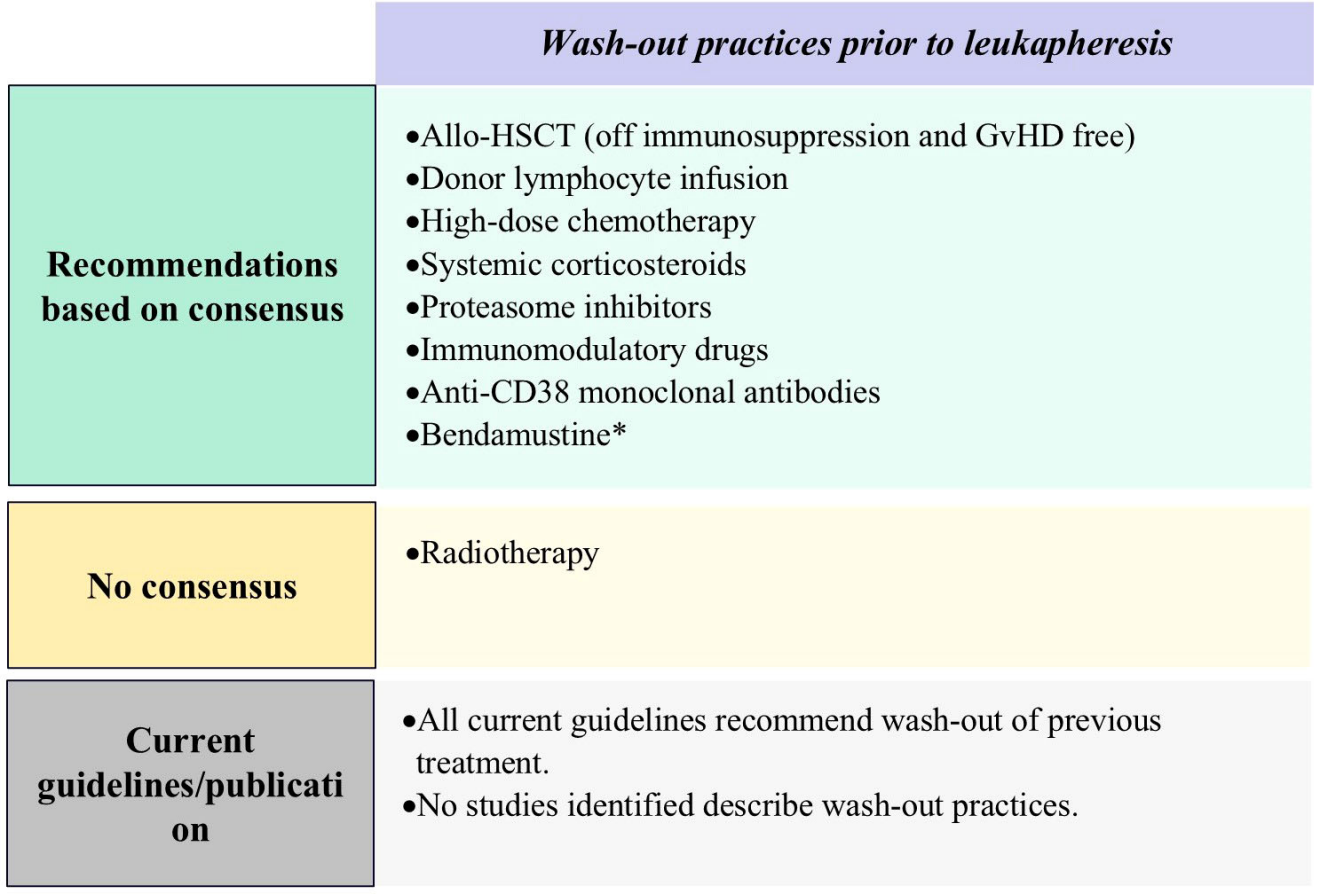
Results of Delphi survey on proposed on wash-out practices. Allo-HSCT, Allogeneic Hematopoietic Stem Cell Transplantation; Anti-CD38, anti-CD38 monoclonal antibody; GvHD, graft-versus-host disease. *Important factors as per panelists’ consensus of agreement.

Panelists agreed that treatments requiring a washout period prior to CAR-T cell infusion should include allo-HSCT (off immunosuppression and absence of GvHD), high-dose chemotherapy, systemic corticosteroids, PIs, IMiDs, donor lymphocyte infusion, and anti-CD38 monoclonal antibodies. Washout periods for PIs, IMiDs, and anti-CD38 monoclonal antibodies should allow sufficient time for hematological recovery. The minimum washout period for donor lymphocyte infusion should be eight weeks and practitioners should ensure the absence of active GvHD. For other agents, the panelists provided varied suggestions for the minimum wash-out period duration (**Figure 4**; **Supplementary Table S9**).

##### 3.2.3.2 Bridging therapy

**Figure 5:**
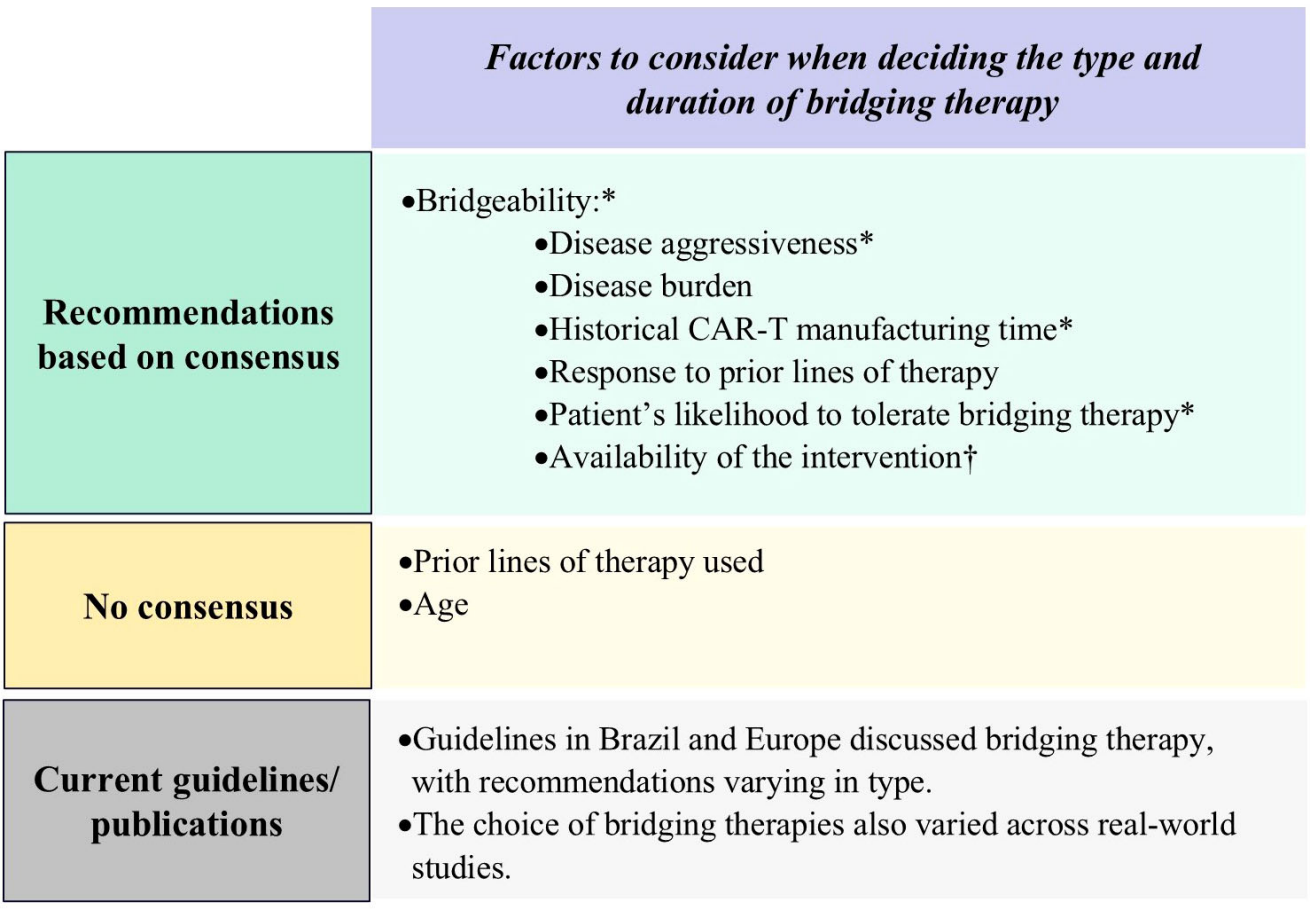
Results of Delphi survey on proposed factors to consider when deciding the type and duration of bridging therapy. CAR-T, chimeric antigen receptor T-cell. *Important factors as per panelists’ consensus of agreement; †Practical factors as per panelists’ consensus of agreement.

To guide the selection of bridging therapy, clinicians should prioritize the following factors in order of importance: kinetics of disease progression, the patient’s likelihood to tolerate bridging therapy, historical CAR-T cell manufacturing time, disease burden, availability of the intervention, and response to prior therapies. These factors collectively reflect what we have defined as a patient’s ‘bridgeability’, which is very important to consider in the choice of bridging therapy for individual patients (**Figure 5**; **Supplementary Table S10**).

Panelists concluded that practitioners should ideally select a regimen for bridging therapy which is effective yet minimally toxic. For patients with low disease burden or slow progression, bridging therapy may not be necessary.

##### 3.2.3.3 Lymphodepletion and infusion

**Figure 6:**
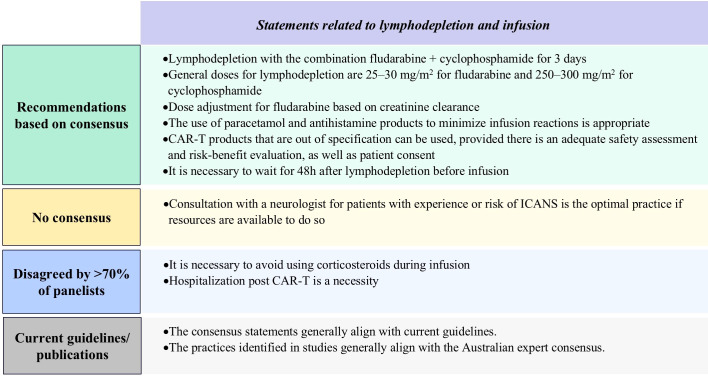
Results of Delphi survey on proposed statements related to lymphodepletion and infusion. CAR-T, chimeric antigen receptor T-cell; ICANS, immune effector cell-associated Neurotoxicity. Syndrome.

Standard lymphodepletion protocols include a 3-day intravenous regimen of 25−30 mg/m² fludarabine and 250−300 mg/m² cyclophosphamide, with fludarabine dose adjustments if appropriate based on renal function. A minimum of 48 hours is required between completion of lymphodepletion and CAR-T cell infusion, with premedication with antihistamine and acetaminophen (paracetamol), as well as corticosteroids if necessitated. While not mandatory, the decision to hospitalize a patient post-infusion should be made with consideration of the median time to onset of adverse events specific to the infused product. If the patient is treated on an outpatient basis there should be an adequate monitoring plan in place, they should reside within an agreed-upon travel time from the hospital, and readmission should be at a prearranged time (as was done in clinical trials) or promptly upon development of symptoms. Practitioners can use CAR-T products that are out of specification, provided there is an adequate safety assessment and risk-benefit evaluation, as well as patient consent. Although 70% consensus was not reached, panelists in general agreed that consultation with neurologists for patients with a history or risk of ICANS is good practice if available (**Figure 6**; **Supplementary Table S11**).

#### 3.2.4 Post-treatment management

**Figure 7:**
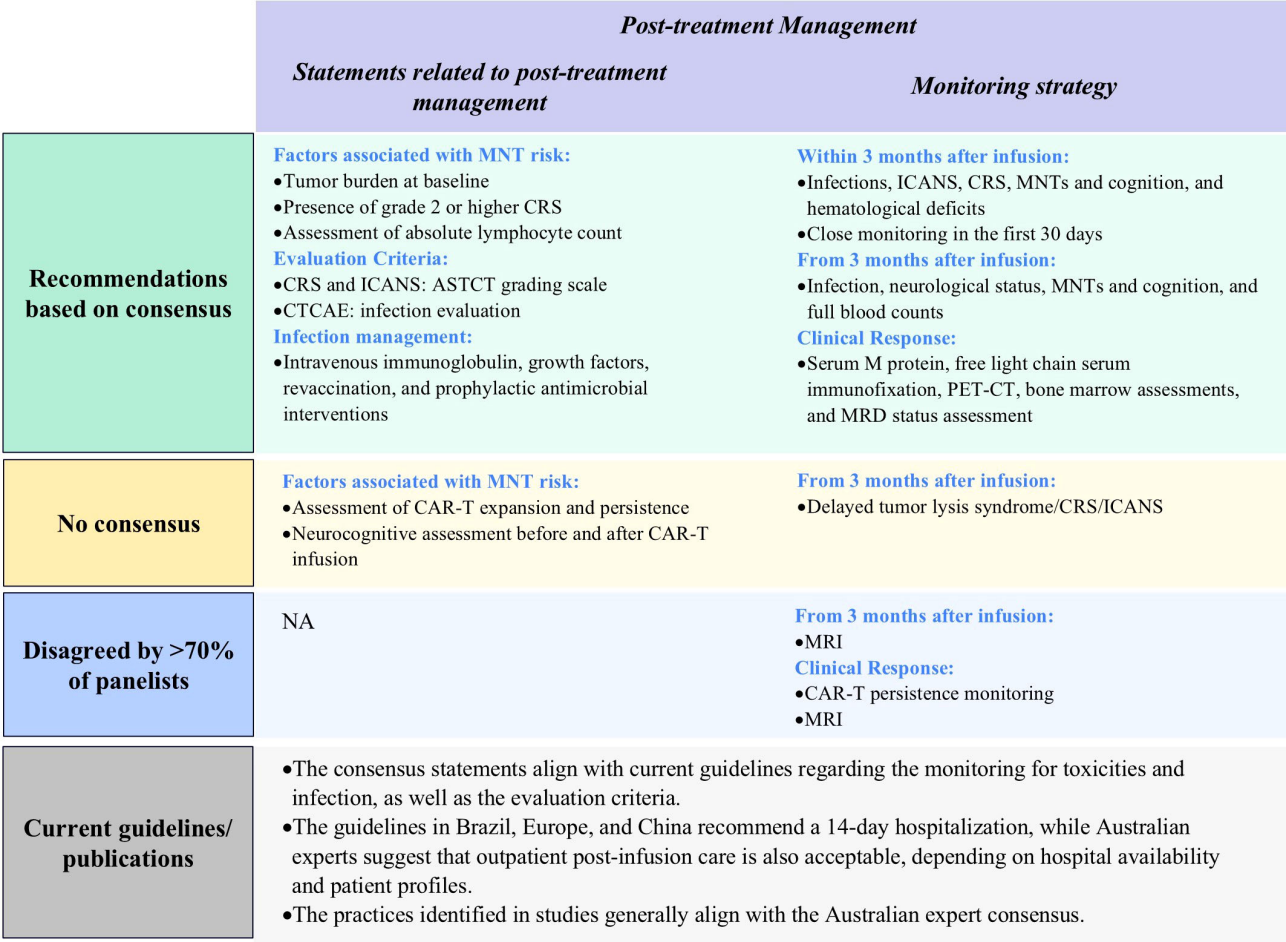
Results of Delphi survey on proposed statements related to post-treatment management. ASTCT, American Society for Transplantation and Cellular Therapy; CAR-T, chimeric antigen receptor T-cell; CRS, Cytokine Release Syndrome; CTCAE, Common Terminology Criteria for Adverse Events; ICANS, Immune Effector Cell-Associated Neurotoxicity Syndrome; MNT, movement and neurocognitive treatment emergent adverse event; MRI, magnetic resonance imaging; NA: not applicable; MRD, minimal residual disease; PET-CT, Positron Emission Tomography-Computed Tomography.

Panelists reached consensus on several facets of post-treatment management. For example, clinicians should assess factors associated with increased risk of movement and neurocognitive treatment-emergent adverse events (MNTs), which include tumor burden at baseline and presence of grade 2 or higher CRS. There is increasing evidence that ALC is correlated with MNT, and observation of ALC in the first 2 weeks post CAR-T infusion should be considered, to determine whether prophylactic measures such as steroids should be administered in the case of high elevations of ALC post infusion (23, 37). Panelists recommended use of the ASTCT grading scale for CRS and ICANS, and Common Terminology Criteria for Adverse Events (CTCAE) for infection evaluation, with inpatient or outpatient post-infusion care dependent on hospital availability and patient profiles. For infection management, the Delphi panel recommended that clinicians use intravenous immunoglobulin, growth factors, revaccination, and prophylactic antimicrobial interventions (**Figure 7**; **Supplementary Table S12**).

##### 3.2.4.1 Monitoring strategies within 30 days post infusion^1^

Panelists agreed that patients should be closely monitored for infections, ICANS, CRS, MNTs, and cognition and hematological deficits after infusion, with higher monitoring frequencies in the first 30 days. Specifically, hospitalized patients should be monitored daily for the first 14 days for CRS, hematological abnormalities, MNTs, and cognitive disfunction. Following this, the monitoring frequency can be reduced to twice per week until 30 days post-infusion (**Figure 7**, **Table 2**; **Supplementary Table S13**).

**Table 2:** Recommendations on monitoring strategies based on consensus of agreement.

Factor	Suggested approach
Within 3 months post-infusion	
CRS	• Daily during first 14 days when hospitalized, then twice per week post discharge for first month, then every 4 weeks until 3 months
Full blood count	• Daily during first 14 days when hospitalized, then twice per week post discharge for first month, then every 4 weeks until 3 months • Every visit for outpatient, or daily for inpatient
MNTs, cognition	• Daily during first 14 days when hospitalized, then twice per week post discharge for the first month, and then every 4 weeks
From 3 months post-infusion	
Neurological status	• Every visit
MNTs, cognition	• Every 3 months
Clinical response	
PET-CT	• As clinically indicated
Bone marrow assessment	• Upon indication, unexplained cytopenia, suspicion of secondary bone marrow malignancies, or progressive disease

CRS, Cytokine Release Syndrome; ICANS, Immune Effector Cell-Associated Neurotoxicity Syndrome; MNT, movement and neurocognitive treatment emergent adverse event; PET-CT, Positron Emission Tomography-Computed Tomography.

##### 3.2.4.2 Monitoring strategies 30 days after infusion

Following the initial 30-day period after infusion, monitoring for CRS and ICANS should be continued with or without reduced frequency depending on clinical status. From three months after CAR-T cell infusion, practitioners should continue to monitor patient neurological and infection status and perform full blood counts. Clinicians should evaluate MNTs and cognition every 3 months (**Figure 7**, **Table 2**; **Supplementary Table S13**).

##### 3.2.4.3 Monitoring for clinical response

Clinical response monitoring for CAR-T cell therapy should include serum M protein quantification, free light chain, serum immunofixation, PET-CT as clinically appropriate, bone marrow assessments (as needed for unexplained cytopenias or disease progression), and minimal residual disease (MRD) status assessment. Panelists did not recommend that clinicians monitor CAR-T cell persistence, emphasizing that this may not be feasible in all Australian settings (**Figure 7**, **Table 2**; **Supplementary Table S13**).

## 4 Discussion

We conducted a Delphi panel to establish consensus on optimal management practices for CAR-T cell therapy for patients in Australia with MM, informed by initial results of our SLR. The SLR highlighted differing recommendations and approaches to patient referral, selection, prioritization, infusion management, and post-infusion monitoring, and no guidelines or recommendations were identified specifically for Australian settings. While the IMWG guidelines were not yet available during the conduct of this panel (20), our comprehensive search and review of available studies during the time-frame provided valuable information on inter-regional differences in recommendations.

### 4.1 Consensus of clinical practice in the Australian setting

To the best of our knowledge, this is the first attempt to develop an expert consensus in Australia for the management of patients with MM being considered for CAR-T cell therapy. The Delphi panel reached consensus on many statements surrounding considerations for patient referral and selection, prioritization, optimal screening practices, treatment management, and post-infusion management.

The Delphi panel achieved consensus on general clinical practices, as well as on specific details such as the timing and cut-off thresholds for adverse events and clinical response assessments to initiate subsequent management processes. Most consensus statements from the Delphi survey aligned with the recommendations in clinical practice guidelines included in the SLR. An exception to this was that panelists agreed that post-infusion hospitalization is not always required, in contrast to recommendations for 14 days admission following infusion in the Brazilian, European, and Chinese guidelines (18, 38, 39).

### 4.2 Divergent opinions and challenges

In our survey, statements with differing opinions in the first round were discussed during the virtual meeting and were addressed in the second round of survey. During this process, consensus on additional statements were reached, while the remaining conflicting opinions were summarized. These differing opinions reflect the uncertainty around the best practices for CAR-T cell therapy for patients with MM, as well as the heterogeneity of Australian clinical practices. For example, regarding optimal timing of referral of patients with RRMM, the panelists discussed whether it is preferable to refer patients before or at the time of progression. Some advocated for “the earlier, the better,” while others noted the potential considerable time lag from progression to becoming eligible, highlighting heterogeneity of clinical practice as well as patient status, and the potential to explore this in further detail.

Another point of contention was the selection criteria for treatment initiation after referral, with lack of consensus on specific thresholds relating to fitness, organ function, and life expectancy. Having a robust patient selection process increases the probability of identifying eligible patients with T-cells that are conducive to the production of efficient autologous CAR-T cells, which in turn may lead to improved health outcomes in the patients who receive therapy (40). However, as demonstrated by this Delphi procedure, determining the exact selection criteria for treatment initiation is challenging, which aligns with previous research from the US (41). Nevertheless, the Delphi process has provided data that would be useful for practitioners (refer to appropriate **Supplementary Material** on the results).

Some conflicting opinions we identified also suggested the challenges or barriers related to CAR-T therapy in Australia. For example, panelists agreed that treatment eligibility criteria based on prior treatment lines should conform with public reimbursement criteria. However, panelists also expressed that these criteria be regularly updated to reflect specific prior classes of therapy as opposed to total prior lines of therapy. Another example was the diverging opinions on neurological consultation before the CAR-T cell infusion. Although less than 70% consensus was reached in the final round of vote, the panelists pointed out during the meeting that while this is the preferred practice, it is not feasible currently due to resource constraints in Australia.

### 4.3 Knowledge gaps and research needs

Further research is needed to address the above non-consensus statements and practice strategies where there was a lack of consensus, such as optimal timing for patient referral and selection criteria. In addition, there are further questions that are worth exploring following this Delphi panel. During the meeting, panelists pointed out that adequate caregiver support is important for CAR-T therapy. Further research is needed to evaluate the role of caregiver support as well as its burden, and to answer the question of how healthcare systems and other stakeholders could help to mitigate the challenges related to caregiver support. Secondly, development of patient-reported outcome measures which could assess the psychosocial impact beyond clinical efficacy may be needed. Thirdly, any potential disparities related to treatment accessibility and equity issues should be considered, especially for patients from rural or remote areas, or socioeconomically vulnerable patients.

Regarding post-infusion treatment, consensus was reached on the use of prophylactic antimicrobial interventions. Further research is needed to explore the detailed recommendations on the best practices for prophylaxis, considering the diversity of practices applied across the healthcare institutions and regions.

Cilta-cel and ide-cel were approved in recent years by the US FDA for the treatment of MM, and the Australian TGA granted approval in 2023. Consequently, real-world data on outcomes of these therapeutics remains limited. Patient referral, selection, and prioritization criteria were based solely on studies conducted in the US and may have limited generalizability to Australia due to differences in resource allocation and value judgments in clinical practice. Consequently, further evidence on the effectiveness of the treatments in real-world settings in Australia is needed to inform appropriate practices, especially for the divergent opinions identified by our Delphi study. Firstly, there were knowledge gaps in some specific patient groups such as elderly and frail patients who were mostly underrepresented in trials. One publication mentioned that most real-world patients in the US did not meet the inclusion criteria for trials, but the efficacy and safety outcomes were still comparable (27). Further observations would be needed in the future to validate the clinical outcomes for specific groups as well as overall cohorts in Australia.

The findings from this Delphi panel study have been developed with the intention of being applied in all regions of Australia, as reflected by the inclusion of representatives from these different regions. That said, further research may be needed to evaluate whether geographic constraints and resource disparities would have an impact on the extent to which consensus statements can be applied to individual regions in Australia, as well as to explore how practices may need to be adapted across the regions.

In addition, while this research has had a specific focus on Australia, the findings from this study may also inform practices and research needs for other countries or regions with similar healthcare challenges, such as countries with diverse healthcare resource levels and patient populations. Further research and expert consensus may be needed on the emerging new treatments such as bispecific antibodies and targeted immune checkpoint inhibitors, to explore the best practices to integrate with CAR-T therapy. Emerging evidence also suggests an association between tumor microenvironment and MM progression through angiogenesis and stromal interactions, and the efficacy of new agents targeting VEGF and HGF (42, 43). Given these agents could disrupt the vascular and growth factor-mediated support that are critical for tumor survival, there could be a potential integration of CAR-T therapy with the new emerging treatments. This in turn could also influence the treatment algorithm of MM globally as well as in Australia.

### 4.4 Strengths and limitations

The design of our Delphi panel study was an adaptation of the RAND/UCLA design, a method which offers several strengths conducive to gathering expert consensus on complex healthcare practices (13, 14, 44). Compared to other social research, such as focus groups or interviews, Delphi study has several strengths. Firstly, the responses were kept anonymous and independent to encourage unbiased input from panelists. The virtual meeting focused on discussion on the insights related to the research questions instead of voting on agreement face-to-face, which could enrich the panelists’ knowledge and allow them to correct any misconceptions in the following round of the survey. Secondly, the method is flexible to allow researchers to adapt the technique to the given context. Further, multiple rounds of survey encouraged the panelists to think about the queries several times, which enhances the validity of the data (45). Participating practitioners were from different Australian states which ensured that the panel recommendations reflected regional differences in healthcare practices and patient populations. A further strength of our Delphi panel study was the high response rate among panelists, with responses received for 88% to 100% of survey questions.

Another strength is that a comprehensive SLR was conducted to inform the earliest statements suggested in the Delphi questionnaires. The SLR used a robust methodology informed by PRISMA guidance to identify all relevant evidence for each step of the management process for patients with MM undergoing CAR-T cell therapy (12). In addition, we identified and combined evidence from clinical trials and real-world studies to provide information on current practices for patient identification, referral/prioritization, and management before, during, and after CAR-T cell infusion.

One possible limitation of the Delphi method we employed was the use of alternative scales (five-point and three-point scales) in our survey, instead of a nine-point scale recommended for RAND/UCLA methodology (13, 14). We decided that the combination of a lower point scale and the definition of agreement would align broadly with the RAND/UCLA method. For example, in our definition of consensus we stipulated that at least 70% of panelists needed to provide the same response on the three-point scale. Studies strictly conforming to RAND/UCLA methodology and using a nine-point scale often define consensus to include a quorum of responses within a three-point range (13, 14). There is little practical difference between the two approaches with likely minimal impact on the outcomes. In addition, the use of alternative scales has been recommended when the objective is to obtain final consensus (15).

Another potential limitation was the definition of consensus used, which was based on 70% agreement as used in De Meyer et al. (2019) (15). This is lower than the 80% level frequently used in studies with Delphi panels. The rationale for a lower threshold was the comparably lower number of panelists which would have rendered a higher threshold impractical since agreement from at least seven of the eight panelists would be required to achieve consensus. Out of the 110 statements tested in this Delphi study, 95 reached consensuses based on the 70% criterion. If an 80% threshold had been applied, 57 statements would still have reached consensus, highlighting that had a higher threshold been used, substantially fewer statements would have been defined as reaching consensus, even though in many cases there was broad agreement from the panelists.

## 5 Conclusion

Our Delphi survey explored critical aspects of CAR-T cell therapy for patients with MM, providing a comprehensive overview of current global practices and the first consensus-driven recommendations for practitioners in Australia. By identifying key areas of patient selection, pre-infusion management, and post-infusion monitoring, we have outlined a framework to guide the development of standardized protocols. The insights from the Delphi panel highlight the importance of practicality and feasibility for implementing CAR-T cell therapy management practices to ensure that they are adaptable to the Australian healthcare context. Our findings will inform the development of Australian clinical practice guidelines and promote collaboration among healthcare providers, researchers, and policymakers, which in turn could optimize the effectiveness and safety of CAR-T cell therapy for patients with MM in Australia. Future research is warranted to improve clarity and understanding on topics where consensus was not reached, particularly regarding the timing of referral, patient selection, and aspects of post-CAR-T therapy monitoring protocols.

## Data Availability

The original contributions presented in the study are included in the article/**Supplementary Material**. Further inquiries can be directed to the corresponding author.

## Footnotes

1 Please note that the Delphi questionnaire originally differentiated between monitoring before and after three months. However, based on the responses and feedback of the panelists, a 30-day period to differentiation the monitoring strategies was regarded as more appropriate.
