# Supplementary material for: Management of CAR-T cell therapy in patients with multiple myeloma: a systematic review and expert consensus in Australia
Source: Front Oncol. 2025 Jan 21;14:1535869. doi: 10.3389/fonc.2024.1535869 (PMC11790593; doi:10.3389/fonc.2024.1535869)
Supplement: Supplementary file 1 [file DataSheet1.pdf]

Questionnaire 1: Panellist expertise

Question 1

Please tick the appropriate box to indicate your primary medical specialty.

☐ Haematology

☐ Oncology

☐ Other (please specify in the comment box) COMMENT

Question 2

Please tick the appropriate box to indicate whether you are specialized in the treatment of MM, or whether you typically refer patients with MM to another specialist for treatment.

Treatment experience

☐ I have direct experience in the treatment of MM.

☐ I typically refer patients with MM to another specialist for treatment.

☐ Other (please specify in the comment box) COMMENT

CAR-T experience

☐ I have direct experience in CAR-T treatment of MM

☐ I typically refer MM patients to another specialist for CAR-T treatment

☐ I have no experience in the treatment of MM patients with CAR-T therapy, but I have experience with other treatments COMMENT

Question 3

Please specify in the comment box the institution/medical centre where you practice your medical specialty.

☐ Royal Brisbane & Women's Hospital

☐ Calvary Mater Newcastle Hospital

☐ Royal Prince Alfred Hospital

☐ Westmead Hospital

☐ Peter MacCallum Cancer Centre

☐ St Vincent's Hospital Melbourne

☐ Austin Hospital

☐ Fiona Stanley Hospital

☐ Royal Children's Hospital

☐ Sydney Children's Hospital

☐ Others (please specify in the comment box) COMMENT

Question 4

Please indicate the average <u>monthly</u> number of patients with **MM** that you would typically manage.

If you would like to provide further clarification, you may include a comment.

NUMBER

COMMENT

Question 5

Please indicate the average <u>monthly</u> number of patients that would be referred for **CAR-T therapy** (both personally and by the whole institution/hospital if available. Please specify if for MM or in general across conditions).

If you would like to provide further clarification, you may include a comment.

MM treated with 3 or more prior lines of therapy	NUMBER	COMMENT
MM treated with 4 or more prior lines of therapy	NUMBER	COMMENT
Acute lymphoblastic lymphoma	NUMBER	COMMENT
Diffuse large-B cell lymphoma	NUMBER	COMMENT
Other diseases	NUMBER	COMMENT

Question 6

Please indicate below the numbers of published scientific manuscripts and/or conference abstracts you co-authored, and clinical trials on **MM** where you acted as an investigator in the last <u>five years (since 2018)</u>:

Peer-reviewed Journal articles	NUMBER
Congress abstracts and/or conference keynotes	NUMBER
Clinical trials in MM as an investigator	NUMBER

Question 7

Please indicate below the numbers of published scientific manuscripts and conference abstracts you co-authored, and clinical trials on **CAR-T therapies** where you acted as an investigator in the last <u>five years (since 2018)</u>:

Peer-reviewed Journal articles	NUMBER

Congress and/or conference keynotes	NUMBER
Clinical trials in CAR-T therapies as an investigator	NUMBER

Questionnaire 2: Delphi Panel

Note:

1. **Some of the following questions would ask you to scale the opinion in terms of importance, and practicality, please refer to the scale chart below for your answers:**

Table 1. Description of scales rating

Rate	1	2	3	4	5
**Scale for importance**	Very important	Somewhat important	Neither important nor unimportant	Somewhat unimportant	Completely unimportant
**Scale for practicality**	Very practical	Somewhat practical	Neither practical nor impractical	Somewhat impractical	Very impractical

2. **The following questions are developed based on a systematic literature review we conduct on the topic, including trials, real-world studies, guidelines/expert consensus from US, Brazil, Europe and China.**

Patient eligibility & referral

Question 1

What are the **suitable eligibility (i.e., inclusion) criteria utilised in clinical practice to consider MM patients for referral to** CAR-T therapy, in terms of disease severity or progression, lines of treatment exposure/refractory status, estimated life expectancy, organ functions, or any other consideration?

You may provide multiple answers, if applicable. Please specify the parameters if chosen in the table below.

Please add a number from the scales presented in Table 1 to reflect the importance and practicality of each option:

Options	Parameter	Cut-off value	Timing of assessment	**Importance** (Add number from 1 = 'very important' to 5 = 'completely unimportant')	**Practicality** (Add number from 1 = 'very practical' to 5 = 'very impractical')
**Patient-related factors:**					
☐ Age	NA	COMMENT	COMMENT	NUMBER	NUMBER
☐ Adequate cardiac function	COMMENT (e.g., NYHA grade for heart failure, LVEF, and others)	COMMENT	COMMENT	NUMBER	NUMBER
☐ Adequate respiratory function	COMMENT	COMMENT	COMMENT	NUMBER	NUMBER
☐ Adequate renal function	COMMENT (e.g., creatinine clearance, and others)	COMMENT	COMMENT	NUMBER	NUMBER
☐ Adequate liver function	COMMENT (e.g., ALT, AST, bilirubin, and others)	COMMENT	COMMENT	NUMBER	NUMBER
Click or tap here to enter text.☐ Absence of active infection	COMMENT (e.g., HIV, HBV, HCV, CMV, EBV, syphilis, bacterial infections)	COMMENT	COMMENT	NUMBER	NUMBER
☐ Expected life expectancy	NA	COMMENT	COMMENT	NUMBER	NUMBER
☐ Socio-economic status	COMMENT (e.g., carer support, ability to travel, and others)	COMMENT	COMMENT	NUMBER	NUMBER
☐ Frailty score	☐ IMWG Frailty Index<br>☐ Revised Myeloma Comorbidity Index (R-MCI)<br>☐ Mayo Frailty Index	COMMENT	COMMENT	NUMBER	NUMBER

Options	Parameter	Cut-off value	Timing of assessment	Importance (Add number from 1 = 'very important' to 5 = 'completely unimportant')	Practicality (Add number from 1 = 'very practical' to 5 = 'very impractical')
	☐ Other validated frailty assessment (specify) COMMENT<br>☐ Other informal frailty assessment (specify) COMMENT				
☐ Other (please specify in the comment box) COMMENT	COMMENT	COMMENT	COMMENT	NUMBER	NUMBER
**Disease-related factors:**					
☐ ECOG score	NA	COMMENT	COMMENT	NUMBER	NUMBER
☐ RRMM	☐ As per IMWG criteria<br>☐ Other criteria (please specify) COMMENT	COMMENT	COMMENT	NUMBER	NUMBER
☐ Absence of extra-medullary disease	COMMENT	COMMENT	COMMENT	NUMBER	NUMBER
☐ Absence of markers of high-risk cytogenetics	COMMENT	COMMENT	COMMENT	NUMBER	NUMBER
☐ Absence of active CNS diseases	COMMENT	COMMENT	COMMENT	NUMBER	NUMBER
☐ Low risk disease	COMMENT(e.g., R-ISS)	COMMENT	COMMENT	NUMBER	NUMBER
☐ Number or prior lines of treatment	COMMENT (e.g., upon meeting reimbursed eligibility, one line prior	COMMENT	COMMENT	NUMBER	NUMBER

Options	Parameter	Cut-off value	Timing of assessment	**Importance**<br>(Add number from 1 = 'very important' to 5 = 'completely unimportant')	**Practicality**<br>(Add number from 1 = 'very practical' to 5 = 'very impractical')
	to meeting reimbursed eligibility, 2 or more prior lines before meeting reimbursed eligibility)				
☐ Prior **exposed** lines of treatment	COMMENT therapy classes if applicable (e.g., PI, IMiD, mAb, and others)	COMMENT	COMMENT	NUMBER	NUMBER
☐ Prior **refractory** lines of treatment	COMMENT therapy classes if applicable (e.g., PI, IMiD, mAb, and others)	COMMENT	COMMENT	NUMBER	NUMBER
☐ Most current clinical response during referral process	COMMENT	COMMENT	COMMENT	NUMBER	NUMBER
☐ Pace of disease progression	COMMENT	COMMENT	COMMENT	NUMBER	NUMBER
☐ Non-secretory MM	COMMENT	COMMENT	COMMENT	NUMBER	NUMBER
☐ Other (please specify in the comment box) COMMENT	COMMENT	COMMENT	COMMENT	NUMBER	NUMBER

Abbreviations: ALT: alanine transaminase; AST: aspartate transaminase; CAR-T: chimeric antigen receptor T; ECOG: Eastern Cooperative Oncology Group; IMiD: immunomodulatory drugs; IMWG: International Myeloma Working Group; LVEF: left ventricular ejection fraction; mAb: monoclonal antibody; MM: multiple myeloma; NYHA: New York Heart Association; RRMM: relapsed and refractory MM; NA: not applicable; PI: proteasome inhibitor.

Question 2

What are the **reasonable exclusion criteria** for CAR-T therapy in patients with MM?

You may provide multiple answers. Please provide a rationale (e.g., cut-off values, time scope, or any other information that is worth noting) to the chosen answer in the comment box, if applicable.

Please add a number from the scale presented in Table 1 to reflect the importance of each option:

Options	Rationale	Importance (Add a number from 1 = 'very important' to 5 = 'completely unimportant')
☐ Active or poorly controlled CNS disorder (including epilepsy, dementia or CNS involved autoimmune disorder)	COMMENT	NUMBER
☐ Active infection with hepatitis virus (HBV, HCV) and/or others (e.g., EBV, CMV)	COMMENT	NUMBER
☐ Human immunodeficiency virus (HIV)	COMMENT	NUMBER
☐ Other active infection (please specify in the comment box)	COMMENT	NUMBER
☐ Live vaccines within 6 weeks of planned CAR-T infusion	COMMENT	NUMBER
☐ Comorbidities conferring an expected life expectancy of < 5 years (e.g. secondary malignancies)	COMMENT	NUMBER
☐ Active uncontrolled graft-versus-host disease	COMMENT	NUMBER
☐ Cardiovascular diseases	COMMENT	NUMBER
☐ Renal dysfunction	COMMENT	NUMBER
☐ Prior BCMA therapy	COMMENT	NUMBER
☐ Prior treatment with allo-HSCT	COMMENT	NUMBER
☐ Complex psychological issues that may impact on compliance or patient safety	COMMENT	NUMBER

Options	Rationale	Importance<br>(Add a number from 1 = 'very important' to 5 = 'completely unimportant')
☐ Other (please specify in the comment box) COMMENT	COMMENT	NUMBER

Question 3

Please select or indicate any recommended **elements considered for prioritisation of referred patients and** describe the criteria for the selected options.

Please add a number from the scales presented in Table 1 to reflect the importance and practicality of each option:

Options	Criteria	Importance<br>(Add number from 1 = 'very important' to 5 = 'completely unimportant')	Practicality<br>(Add number from 1 = 'very practical' to 5 = 'very impractical')
**Patient-related factors:**			
☐ Age	COMMENT	NUMBER	NUMBER
☐ Disease burden	COMMENT	NUMBER	NUMBER
☐ Time spent on the waitlist	COMMENT	NUMBER	NUMBER
☐ Disease refractoriness	COMMENT	NUMBER	NUMBER
☐ Disease aggressiveness	COMMENT	NUMBER	NUMBER
☐ Likelihood of achieving clinical response	COMMENT	NUMBER	NUMBER
☐ Prior stem cell transplantation	COMMENT	NUMBER	NUMBER

Options	Criteria	**Importance**<br>(Add number from 1 = 'very important' to 5 = 'completely unimportant')	**Practicality**<br>(Add number from 1 = 'very practical' to 5 = 'very impractical')
☐ Haematopoietic Cell Transplantation Comorbidity Index	COMMENT	NUMBER	NUMBER
☐ Other (please specify in the comment box) COMMENT	COMMENT	NUMBER	NUMBER
**System/social-related factors:**			
☐ System/regional capacity	COMMENT	NUMBER	NUMBER
☐ Geographical location limitations	COMMENT	NUMBER	NUMBER
☐ Equity and equality considerations	COMMENT	NUMBER	NUMBER
☐ Availability of alternative treatment options	COMMENT (e.g. non-CAR-T clinical trials)	NUMBER	NUMBER
☐ Other (please specify in the comment box) COMMENT	COMMENT	NUMBER	NUMBER

Question 4

What are the necessary screening practices for patient assessment at the CAR-T treatment centres, for patients who have been referred for CAR-T treatment?

You may provide multiple answers, if applicable.

Options	Parameter	Cut-off value	Timing of assessment
☐ Full Blood count	Haemoglobin	COMMENT	COMMENT
	Platelets	COMMENT	COMMENT
	Lymphocytes	COMMENT	COMMENT
☐ Full biochemistry	Aspartate aminotransferase	COMMENT	COMMENT
	Alanine aminotransferase	COMMENT	COMMENT
	Creatinine clearance	COMMENT	COMMENT
	Total bilirubin	COMMENT	COMMENT
	Corrected serum calcium	COMMENT	COMMENT
☐ Neurological investigations	MRI<br>Electroencephalography (EEG)<br>Lumbar puncture	COMMENT	COMMENT
☐ Adequate cardiac function	COMMENT (e.g., NYHA grade for heart failure, LVEF, and others)	COMMENT	COMMENT
☐ Adequate respiratory function	COMMENT	COMMENT	COMMENT

Options	Parameter	Cut-off value	Timing of assessment
☐ Pregnancy test	NA	Negative/Positive	COMMENT
☐ Whole-body MRI, CT or PET/CT	Detection of possible cancer lesions, neurological and cardiovascular disease	COMMENT	COMMENT
☐ Other (please specify in the comment box) COMMENT	COMMENT	COMMENT	COMMENT

Abbreviations: CT: computed tomography LVEF: left ventricular ejection fraction; mAb: monoclonal antibody; MRI: magnetic resonance imaging; NYHA: New York Heart Association; PET/CT: positron emission tomography-computed tomography.

Note: testing for infectious diseases is mandated by TGA, for which survey of consensus would be not necessary, therefore not listed in this questionnaire.

Pre-CAR-T management and infusion

Question 5

Please select from the table below whether any wash-out periods from prior procedures must be observed prior to **leukapheresis**.

Please also indicate the recommended minimal timeframe for the selected treatments and **add a number from the scale presented in Table 1 to reflect the importance of each selected option:**

Options	Minimal wash-out timeframe	Importance<br>(Add number from 1 = 'very important' to 5 = 'completely unimportant')
☐ Allo-HSCT (off immunosuppression and GvHD free)	☐ 3 to 7 days<br>☐ 2 weeks<br>☐ 3 weeks<br>☐ 4 weeks<br>☐ 8 weeks<br>☐ Other COMMENT	NUMBER

Options	Minimal wash-out timeframe	**Importance**<br>(Add number from 1 = 'very important' to 5 = 'completely unimportant')
☐ Donor lymphocyte infusion	☐ 3 to 7 days<br>☐ 2 weeks<br>☐ 3 weeks<br>☐ 4 weeks<br>☐ 8 weeks<br>☐ Other COMMENT	NUMBER
☐ CNS radiotherapy	☐ 3 to 7 days<br>☐ 2 weeks<br>☐ 3 weeks<br>☐ 4 weeks<br>☐ 8 weeks<br>☐ Other COMMENT	NUMBER
☐ High-dose chemotherapy	☐ 3 to 7 days<br>☐ 2 weeks<br>☐ 3 weeks<br>☐ 4 weeks<br>☐ 8 weeks<br>☐ Other COMMENT	NUMBER
☐ Systemic corticosteroids	☐ 3 to 7 days<br>☐ 2 weeks	NUMBER

Options	Minimal wash-out timeframe	**Importance**<br>(Add number from 1 = 'very important' to 5 = 'completely unimportant')
	☐ 3 weeks<br>☐ 4 weeks<br>☐ 8 weeks<br>☐ Other COMMENT	
☐ Proteasome inhibitors	☐ 3 to 7 days<br>☐ 2 weeks<br>☐ 3 weeks<br>☐ 4 weeks<br>☐ 8 weeks<br>☐ Other COMMENT	NUMBER
☐ Immunomodulatory drugs	☐ 3 to 7 days<br>☐ 2 weeks<br>☐ 3 weeks<br>☐ 4 weeks<br>☐ 8 weeks<br>☐ Other COMMENT	NUMBER
☐ Anti-CD38 monoclonal antibodies	☐ 3 to 7 days<br>☐ 2 weeks<br>☐ 3 weeks<br>☐ 4 weeks	NUMBER

Options	Minimal wash-out timeframe	**Importance**<br>(Add number from 1 = 'very important' to 5 = 'completely unimportant')
	☐ 8 weeks<br>☐ Other COMMENT	
☐ Other (please specify in the comment box) COMMENT	☐ 3 to 7 days<br>☐ 2 weeks<br>☐ 3 weeks<br>☐ 4 weeks<br>☐ 8 weeks<br>☐ Other COMMENT	NUMBER

Question 6

The aim of bridging therapy is to reduce tumour burden and maintain performance status before CAR-T infusion.

Please answer the following question around what the **optimal practices for bridging therapy are before initiating lymphodepletion** as part of the treatment plan.

Questions	Answer	If "Yes", please provide a rational in the comment box
Are there specific patient subgroups or are there specific circumstances that bridging therapy may not be necessary or beneficial?	☐ Yes<br>☐ No	COMMENT
Are there any interventions that should be avoided as bridging therapy?	☐ Yes<br>☐ No	COMMENT

Questions	Answer	If “Yes”, please provide a rational in the comment box
Is washout period for bridging therapy necessary?<br>If so, please indicate an appropriate timeframe	☐ Yes<br>☐ No	COMMENT
Could therapies that patients are naïve to be used in bridging therapy?	☐ Yes<br>☐ No	COMMENT

What are the factors taken into account when deciding the type and duration of bridging therapy?

Please add a number from the scales presented in Table 1 to reflect the importance and practicality of each option:

Options	Criteria	**Importance**<br>(Add number from 1 = ‘very important’ to 5 = ‘completely unimportant’)	**Practicality**<br>(Add number from 1 = ‘very practical’ to 5 = ‘very impractical’)
☐ Age	COMMENT	NUMBER	NUMBER
☐ Disease burden	COMMENT	NUMBER	NUMBER
☐ Historical CAR-T manufacturing time	COMMENT	NUMBER	NUMBER
☐ Disease refractoriness	COMMENT	NUMBER	NUMBER
☐ Disease aggressiveness	COMMENT	NUMBER	NUMBER
☐ Prior lines of therapy used	COMMENT (e.g., only agents that have been used before can be used for bridging)	NUMBER	NUMBER
☐ Response to prior lines of therapy	COMMENT	NUMBER	NUMBER
☐ Patient’s likelihood to tolerate bridging therapy	COMMENT	NUMBER	NUMBER

Options	Criteria	**Importance**<br>(Add number from 1 = 'very important' to 5 = 'completely unimportant')	**Practicality**<br>(Add number from 1 = 'very practical' to 5 = 'very impractical')
☐ Availability on the PBS	COMMENT	NUMBER	NUMBER
☐ Other (please specify in the comment box) COMMENT	COMMENT	NUMBER	NUMBER

If any further recommendations, please provide in the following comment box:

COMMENT

Question 7

What are the necessary and appropriate **practices for lymphodepletion**?

You may provide multiple answers. Please provide a rationale (e.g., detailed practices, timelines, or any other information that worth noting) to the chosen answer in the comment box, if applicable.

Efficacy related practices for successful lymphodepletion:

☐ Lymphodeplete patients with the combination fludarabine / cyclophosphamide for 3 days COMMENT

☐ General doses for lymphodepletion are 25-30 mg/m^2 for fludarabine and 250-300 mg/m^2 for cyclophosphamide COMMENT

☐ Other (please specify in the comment box) COMMENT

Safety related practices for successful lymphodepletion:

☐ Consultation with a neurologist for patients with experience or risk of ICANS COMMENT

☐ Dose adjustment for fludarabine based on creatinine clearance COMMENT

☐ Other (please specify in the comment box) COMMENT

Question 8

Please answer the following questions around the **best practices for CAR-T infusion**

Please provide a rationale (e.g., detailed practices, timelines, or any other information that is worth noting) if applicable.

Questions	Answer	If "Yes", please provide a rational in the comment box
**Efficacy related practices**:		
Is it necessary to wait for a specific number of days after lymphodepletion before infusion initiated?<br>If so, indicate an appropriate duration time.	☐ Yes<br>☐ No	COMMENT
**Safety related practices**:		
Is hospitalisation post CAR-T a must?<br>If so, on what day and what is the expected duration?	☐ Yes<br>☐ No	COMMENT
Is it an appropriate practice to use paracetamol and antihistamine products to minimize infusion reaction?	☐ Yes<br>☐ No	COMMENT
Is it necessary to avoid using corticosteroids during infusion?	☐ Yes<br>☐ No	COMMENT
If out-of-specification CAR-T product available, would you agree to use it?<br>If so, what are the necessary practices?	☐ Yes<br>☐ No	COMMENT

If any further recommendations, please provide in the following comment box:

COMMENT

Post CAR-T management

Question 9

What are the necessary **practices after CAR-T therapy infusion**?

You may provide multiple answers. Please provide a rationale (e.g., detailed practices, timelines, or any other information that worth noting) to the chosen answer in the comment box, if applicable.

☐ Toxicity monitoring including cytokine release syndrome (CRS) and neurological events (please also specify the **frequency and timeframe of follow up**) COMMENT

☐ Early administration of tocilizumab for patients with CRS (please also describe in which cases this applies) COMMENT

☐ Regular monitoring for the following: (please also specify the **frequency of monitoring** in the comment box)

- ☐ Cytopenia COMMENT
- ☐ Infectious events COMMENT
- ☐ Organ function COMMENT
- ☐ Other (please specify in the comment box) COMMENT

☐ Assessment of factors associated with increased risk for movement and neurocognitive treatment-emergent adverse events (MNT):

- ☐ Assessment of tumour burden at baseline COMMENT
- ☐ Presence of grade $\geqslant$ 2 CRS COMMENT
- ☐ Assessment of CAR T expansion and persistence COMMENT
- ☐ Assessment of absolute lymphocyte count on days 14, 21 and 28 post-CAR T infusion COMMENT
- ☐ Other (please specify in the comment box) COMMENT

☐ Management of infection (please also specify the regimen of the selected treatment, e.g., dose, administration schedule, duration, etc.):

- ☐ Prophylactic antimicrobial interventions COMMENT
- ☐ Use of intravenous immune globulin therapy COMMENT
- ☐ Revaccination COMMENT
- ☐ Use of growth factor support COMMENT
- ☐ Other (please specify in the comment box) COMMENT

☐ Tumour sample testing to investigate the presence of lentiviral elements if a secondary primary malignancy developments.

Please indicate the grading criteria/scales you recommend for monitoring the conditions below:

Conditions	Evaluation criteria
CRS	☐ ASTCT Grading scale for CRS<br>☐ Penn grading scale<br>☐ CTCAE<br>☐ Other COMMENT
Immune effector cell–associated neurotoxicity syndrome (ICANS)	☐ ASTCT Grading scale for ICANS.<br>☐ Other COMMENT
Infectious condition	☐ CTCAE<br>☐ Other COMMENT
Organ function	COMMENT
Other (please specify in the comment box) COMMENT	COMMENT

Abbreviations ASTCT: American Society for Transplantation and Cellular Therapy; CTCAE Common Terminology Criteria for Adverse Events; LODS: Logistic Organ Dysfunction Score; MODS: Multiorgan Dysfunction Score; SOFA: Sequential Organ Failure Assessment Score

Question 10

Please outline the recommended **monitoring schemes** for adverse events and disease progression associated with subsequent anti-myeloma therapy. Specify the tests and parameters used, the duration of short-term and long-term monitoring, and the frequency of assessments.

Timeframe	Tests/parameters	Timeframes	Frequency of monitoring
Acute toxicity monitoring	☐ CRS COMMENT	☐ 0 to 14 days<br>☐ 0 to 30 days<br>☐ 0 to 50 days<br>☐ 0 to 100 days<br>☐ Others COMMENT	☐ Every day<br>☐ Every 2 days<br>☐ Every week<br>☐ Every 2 weeks<br>☐ Others COMMENT
	☐ ICANS COMMENT	☐ 0 to 14 days<br>☐ 0 to 30 days<br>☐ 0 to 50 days<br>☐ 0 to 100 days<br>☐ Others COMMENT	☐ Every day<br>☐ Every 2 days<br>☐ Every week<br>☐ Every 2 weeks<br>☐ Others COMMENT
	☐ Infections COMMENT	☐ 0 to 14 days<br>☐ 0 to 30 days<br>☐ 0 to 50 days<br>☐ 0 to 100 days<br>☐ Others COMMENT	☐ Every day<br>☐ Every 2 days<br>☐ Every week<br>☐ Every 2 weeks<br>☐ Others COMMENT

	☐ Full blood count COMMENT	☐ 0 to 14 days<br>☐ 0 to 30 days<br>☐ 0 to 50 days<br>☐ 0 to 100 days<br>☐ Others COMMENT	☐ Every day<br>☐ Every 2 days<br>☐ Every week<br>☐ Every 2 weeks<br>☐ Others COMMENT
	☐ Others COMMENT	☐ 0 to 14 days<br>☐ 0 to 30 days<br>☐ 0 to 50 days<br>☐ 0 to 100 days<br>☐ Others COMMENT	☐ Every day<br>☐ Every 2 days<br>☐ Every week<br>☐ Every 2 weeks<br>☐ Others COMMENT
Chronic toxicity monitoring	☐ Infections COMMENT	☐ 1 years<br>☐ 2 years<br>☐ 5 years<br>☐ Others COMMENT	☐ Every month<br>☐ Every 2 months<br>☐ Others COMMENT
	☐ Full blood count COMMENT	☐ 1 years<br>☐ 2 years<br>☐ 5 years<br>☐ Others COMMENT	☐ Every month<br>☐ Every 2 months<br>☐ Others COMMENT
	☐ MRI COMMENT	☐ 1 years<br>☐ 2 years<br>☐ 5 years<br>☐ Others COMMENT	☐ Every month<br>☐ Every 2 months<br>☐ Others COMMENT
	☐ Neurological status COMMENT	☐ 1 years	☐ Every month

		☐ 2 years<br>☐ 5 years<br>☐ Others COMMENT	☐ Every 2 months<br>☐ Others COMMENT
	☐ Delayed tumour lysis syndrome/CRS/ICANS COMMENT	☐ 1 years<br>☐ 2 years<br>☐ 5 years<br>☐ Others COMMENT	☐ Every month<br>☐ Every 2 months<br>☐ Others COMMENT
	☐ Others COMMENT	☐ 1 years<br>☐ 2 years<br>☐ 5 years<br>☐ Others COMMENT	☐ Every month<br>☐ Every 2 months<br>☐ Others COMMENT
Clinical response	☐ Serum M protein quantitation COMMENT	☐ 5 years<br>☐ 10 years<br>☐ 15 years<br>☐ lifetime<br>☐ Others COMMENT	☐ Every month<br>☐ Every 2 months<br>☐ Every 3 months<br>☐ Every 6 months<br>☐ Others COMMENT
	☐ Serum free light chain COMMENT	☐ 5 years<br>☐ 10 years<br>☐ 15 years<br>☐ lifetime<br>☐ Others COMMENT	☐ Every month<br>☐ Every 2 months<br>☐ Every 3 months<br>☐ Every 6 months<br>☐ Others COMMENT
	☐ Serum immunofixation COMMENT	☐ 5 years<br>☐ 10 years	☐ Every month<br>☐ Every 2 months

	☐ 15 years<br>☐ lifetime<br>☐ Others COMMENT	☐ Every 3 months<br>☐ Every 6 months<br>☐ Others COMMENT
☐ Bone marrow cytology COMMENT	☐ 5 years<br>☐ 10 years<br>☐ 15 years<br>☐ lifetime<br>☐ Others COMMENT	☐ Every month<br>☐ Every 2 months<br>☐ Every 3 months<br>☐ Every 6 months<br>☐ Others COMMENT
☐ Minimal residual disease (MRD) status COMMENT	☐ 5 years<br>☐ 10 years<br>☐ 15 years<br>☐ lifetime<br>☐ Others COMMENT	☐ Every month<br>☐ Every 2 months<br>☐ Every 3 months<br>☐ Every 6 months<br>☐ Others COMMENT
☐ MRI COMMENT	☐ 5 years<br>☐ 10 years<br>☐ 15 years<br>☐ lifetime<br>☐ Others COMMENT	☐ Every month<br>☐ Every 2 months<br>☐ Every 3 months<br>☐ Every 6 months<br>☐ Others COMMENT
☐ PET-CT COMMENT	☐ 5 years<br>☐ 10 years<br>☐ 15 years<br>☐ lifetime<br>☐ Others COMMENT	☐ Every month<br>☐ Every 2 months<br>☐ Every 3 months<br>☐ Every 6 months<br>☐ Others COMMENT

☐ Standard follow-up (e.g., biochemistry panel/AST, ALT, bilirubin, lactate dehydrogenase, etc) COMMENT	☐ 5 years<br>☐ 10 years<br>☐ 15 years<br>☐ lifetime<br>☐ Others COMMENT	☐ Every month<br>☐ Every 2 months<br>☐ Every 3 months<br>☐ Every 6 months<br>☐ Others COMMENT
☐ CAR-T monitoring COMMENT	☐ 5 years<br>☐ 10 years<br>☐ 15 years<br>☐ lifetime<br>☐ Others COMMENT	☐ Every month<br>☐ Every 2 months<br>☐ Every 3 months<br>☐ Every 6 months<br>☐ Others COMMENT
☐ Others COMMENT	☐ 5 years<br>☐ 10 years<br>☐ 15 years<br>☐ lifetime<br>☐ Others COMMENT	☐ Every month<br>☐ Every 2 months<br>☐ Every 3 months<br>☐ Every 6 months<br>☐ Others COMMENT

If you would like to provide further clarification, you may include a comment.

COMMENT
