# Supplementary material for: Management of CAR-T cell therapy in patients with multiple myeloma: a systematic review and expert consensus in Australia
Source: Front Oncol. 2025 Jan 21;14:1535869. doi: 10.3389/fonc.2024.1535869 (PMC11790593; doi:10.3389/fonc.2024.1535869)
Supplement: Supplementary file 2 [file DataSheet2.pdf]

In this round of the Delphi questionnaire, you will be asked to review the responses of the initial survey and virtual meeting and state the extent to which you agree with the given responses.

For each question, a summary of results from the initial survey and discussion points from the virtual meeting are provided in **blue boxes**. Factors/statements that reached consensus in the initial survey are summarized in a **green box**. For this Delphi panel, consensus is defined as **>70% or ≥5/7 respondents** agree or disagree with the statements[1].

The factors/statements that reached consensus in terms of overall agreement, but need further discussion in terms of importance, practicality, and detailed information (for example, cut-off values, timeframes for assessment, etc.) are included in this questionnaire. Factors for which consensus was not reached based on the first round are also included.

For each factor/statement included in this questionnaire, please **indicate whether you agree or disagree in the box next to the statement.**

<u>You may respond with the same answer you previously provided or change your answer in consideration of the outcomes of the initial survey and virtual meeting.</u>

Note: Some of the questions ask you to provide a rating of the degree of importance for the individual response, as well as a rating of how practical the factor would be to implement.[2] A summary of the ratings that can be provided and what each refers to is provided below.

Table 1. Description of scales rating

Rate	1	2	3
**Scale for importance**	Very important	Somewhat important	Not important
**Scale for practicality**	Very practical	Somewhat practical	Impractical

[1] De Meyer, Dorien, et al. "Delphi procedure in core outcome set development: rating scale and consensus criteria determined outcome selection." Journal of Clinical Epidemiology 111 (2019): 23-31.

[2] Please note panellists were asked to rank "practicality" in the first round, and this has been replaced by "ease of implementation" in this round.

Part 1: Patient eligibility & referral

Question 1: What are the **suitable eligibility (i.e., inclusion) criteria** of MM patients **for referral to** CAR-T therapy?

Summary of initial survey:

Consensus (≥5 out of 7 panellists) was reached on **10 factors** being suitable inclusion criteria for referral (See green box below).

Consensus was also reached on age (2/7), the most current clinical response during referral process (1/7), absence of markers of high-risk cytogenetics (1/7), and absence of extra-medullary disease (1/7) **not being relevant for consideration as inclusion criteria**.

No consensus was reached on the **absence of active infection**, **socio-economic status**, **absence of active CNS disease,** and **prior refractory lines of treatment**. In addition, one panellist also suggested that **baseline cognitive status and availability of effective bridging therapy if this is required,** should be considered as inclusion criteria.

Summary of virtual meeting:

- The criteria for referral would be different from the criteria CAR-T therapy initiation in terms of **active infection**. In particular, patients with active infection should not initiate CAR-T therapy. But patients with **active infections can be referred if the infections could be controlled at the time of treatment.** This factor could be added in the referral phase with proper qualification. (Please note that this factor has also been added to Q3 for prioritization based on the discussion during the meeting.)
- 'Socio-economic status' will be rephrased as '**available social support**'. Panellists agreed that patients need to have social support, which is essential at the time of treatment, but is **inappropriate** as solid **inclusion/exclusion criteria for referral**. Instead, this could be a **potential exclusion criterion for initiating treatment** after a patient has been referred, so that it forces a solution to be found for ensuring the individual has some access to carer support once the treatment has started. (Please note that this factor has also been added to Q3 for prioritization based on the discussion during the meeting.)
- Panellists had differing opinions regarding the '**absence of active CNS diseases' criterion**. Two panellists agreed to rephrase this as 'absence of active uncontrolled disease'. Two panellists noted that given the safety consideration for an active but controlled CNS disease, it would be important to stay conservative on this criterion in the context of guidelines. One panellist pointed out that **having it as specific referral criteria may be not appropriate**.

- '**Prior refractory lines of treatment**' is a criterion that would be set in the treatment approval process, therefore it might **not be relevant as a referral criterion and should not be included.**
- Panellists agreed that patients should be **referred as early as possible** and re-screened later in the process if necessary.
- Panellists agreed that **'baseline cognitive status**' and '**availability of effective bridging therapy if this is required**' are more relevant to decision for the treatment centres later, and it is better **not be included as referral criteria.**
- Although consensus was reached that **age** is not a relevant factor for referral, **three panellists mentioned it should be a factor for eligibility during the meeting** while discussing Q3 (prioritisation criteria) and Q6 (factors for bridging).

Q1 statements with consensus (>70% or ⩾5/7 panellists):

- Relevant patient-related factors as referral criteria: adequate cardiac function (7/7), expected life expectancy (6/7), adequate respiratory function (6/7), frailty score (5/7), adequate renal function (5/7), adequate liver function (5/7).
- Relevant disease-related factors as referral criteria: ECOG score (7/7), pace of disease progression (6/7), meet criteria for RRMM (6/7), number of prior lines of treatment (6/7)

Please review the results of the initial survey indicated by **blue columns** in the table below. In the same table, indicate your opinion on **agreement (Y/N)** for the non-consensus factors from the initial survey, as well as **importance and practicality (1, 2 or 3)** for each factor with which you agree.

You may keep the same answer you previously provided or change your answer in consideration of the outcomes of the initial survey and virtual meeting.

Please add a number from the scales presented in Table 1 to reflect the importance and practicality of each option:

Factors	Results of agreement in initial survey (number of panellists)	Agreement (Yes/No)	Results of importance in initial survey* (number of panellists)		Importance (Add number from 1 = 'very important' to 3 = 'not important')	Results of practicality in initial survey* (number of panellists selected)		Practicality (Add number from 1 = 'very practical' to 3 = 'impractical')
			Important	Very important		Practical	Very practical	
**Patient-related factors:**								
Adequate cardiac function	Consensus reached		2	2	1/2/3	1	4	1/2/3
Adequate respiratory function	Consensus reached		3	1	1/2/3	0	5	1/2/3
Adequate renal function	Consensus reached		2	2	1/2/3	2	3	1/2/3
Adequate liver function	Consensus reached		1	3	1/2/3	1	2	1/2/3
Expected life expectancy	Consensus reached		1	2	1/2/3	2	2	1/2/3
Frailty score	Consensus reached		0	3	1/2/3	0	2	1/2/3
Absence of active/uncontrolled infection	4/7	Y/N	0	3	1/2/3	2	1	1/2/3
Available social support	Newly suggested factor during meeting	Y/N	NA		1/2/3	NA		1/2/3

Factors	**Results of agreement in initial survey** (number of panellists)	**Agreement (Yes/No)**	**Results of importance in initial survey*** (number of panellists)		**Importance** (Add number from 1 = 'very important' to 3 = 'not important')	**Results of practicality in initial survey*** (number of panellists selected)		**Practicality** (Add number from 1 = 'very practical' to 3 = 'impractical')
			**Important**	**Very important**		**Practical**	**Very practical**	
Baseline cognitive status	Newly suggested by one panellist in first round	Y/N	0	1	1/2/3	0	1	1/2/3
Age	2/7 during first round, but multiple panellists mentioned as relevant during meeting in separate discussion	Y/N	0	2	1/2/3	1	1	1/2/3
**Disease-related factors:**								
ECOG score	Consensus reached		1	3	1/2/3	1	3	1/2/3
Meet criteria of RRMM	Consensus reached		0	3	1/2/3	0	3	1/2/3
Pace of disease progression	Consensus reached		1	3	1/2/3	1	3	1/2/3
Prior **exposed** lines of treatment	Consensus reached		1	0	1/2/3	0	1	1/2/3
Absence of active/uncontrolled CNS diseases	4/7	Y/N	0	3	1/2/3	0	2	1/2/3
Prior **refractory** lines of treatment	3/7	Y/N	0	0	1/2/3	1	0	1/2/3
Availability of effective bridging therapy if this is required	Newly suggested by one panellist in first round	Y/N	0	1	1/2/3	0	1	1/2/3

Abbreviations: CAR-T: chimeric antigen receptor T; CNS: central nervous system; ECOG: Eastern Cooperative Oncology Group; RRMM: relapsed and refractory MM; Y/N: yes/no.

**Only 4/7 panellists provided scores for importance and practicality based on the scale defined in the initial survey.*

If you have any further recommendations or clarifications, please provide these in the following comment box:

COMMENT

In the initial survey, panellists provided answers related to what **criteria or cut-off values** should be considered for the **factors that should be included from the above table. In the table below,** please review the responses and **indicate the one(s) with which you agree (Y/N)**.

Please note, if in the **previous table you have not selected that you agree** with the factors being part of the inclusion criteria, **please do not provide an answer for this factor in the table below.**

You may keep the same answer you previously provided or change your answer in consideration of the outcomes of the initial survey and virtual meeting.

Factors	Criteria/cut-off suggested in the initial survey (number of panellists)	Agreement (Yes/No), please leave the cell blank if in the previous table you stated the corresponding factor should not be included at all
**Patient-related factors:**		
Adequate cardiac function	LVEF >40% (3/7)	Y/N
	LVEF >45% (1/7)	Y/N
	LVEF >50% (2/7)	Y/N
	NYHA grade<2 + LVEF>40% (1/7)	Y/N
Adequate respiratory function	None home-O_2 required (1/6)	Y/N
	Oxygen saturation>90% (2/6)	Y/N
	FEV and DLCO>50% (1/6)	Y/N
	DLCO>40% (1/6)	Y/N
Adequate renal function	Creatinine clearance >30ml/min (2/5)	Y/N
	Creatinine clearance >40ml/min (2/5)	Y/N
	eGFR>30 (1/5)	Y/N
Adequate liver function	ALT/AST <5 x ULN, total bilirubin <2 x ULN (2/5)	Y/N
	ALT/AST <3 x ULN, total bilirubin <3 x ULN (1/5)	Y/N
Expected life expectancy	>5 years (3/6)	Y/N
	>6 months (1/6)	Y/N
	>3 months (1/6)	Y/N
Frailty score	ECOG performance status 0-1 (1/5)	Y/N
	IMWG Frailty Index as frail (2/5)	Y/N
	Simplified Frailty Score≥2 (1/5)	Y/N
Absence of active/uncontrolled infection	HIV, HBV, HCV, CMV, EBV, syphilis, bacterial infections (2/4)	Y/N
	As per TGA (1/4)	Y/N
Baseline cognitive status	MoCA<25 (1/1)	Y/N

Factors	Criteria/cut-off suggested in the initial survey (number of panellists)	Agreement (Yes/No), **please leave the cell blank if in the previous table you stated the corresponding factor should not be included at all**
**Disease-related factors:**		
ECOG score	<2 (5/7)	Y/N
	<3 (1/7)	Y/N
Meet criteria of RRMM	As per IMWG criteria (2/5)	Y/N
	As per MSAC wording (1/5)	Y/N
Pace of disease progression	Ability to bridge therapy (3/6)	Y/N
	Ability to infusion with controlled disease (1/6)	Y/N
	Non-rampant progression based on LDH, IMWG criteria, proliferation index, plasmablastic morphology (1/6)	Y/N
Prior **exposed** lines of treatment	3 classes (1/5)	Y/N
	3 lines (1/5)	Y/N
	1 line (1/5)	Y/N
	As per reimbursement criteria/MSAC wording (2/5)	Y/N
Absence of active CNS diseases	MRI and LP (1/4)	Y/N
	MRI and CSF (1/4)	Y/N
Prior **refractory** lines of treatment	Refractory to 1 drug in each class (3 classes) (1/3)	Y/N
	As per reimbursement criteria (1/3)	Y/N

Abbreviations: ALT/AST: alanine aminotransferase / aspartate aminotransferase; CMV: cytomegalovirus; CNS: central nervous system; CSF: cerebrospinal fluid; DLCO: Lung Diffusion Testing; EBV: Epsteine-Barr virus; ECOG: Eastern Cooperative Oncology Group; eGFR: Estimated glomerular filtration rate; FEV: Forced expiratory volume; HBV: hepatitis B virus; HCV: hepatitis C virus; HIV: human immunodeficiency virus; IMWG: International Myeloma Working Group; LDH: Lactate dehydrogenase; LP: lumbar puncture; LVEF: Left ventricular ejection fraction; MRI: Magnetic resonance imaging; MoCA: Montreal Cognitive Assessment; MSAC: Medical and Scientific Advisory Council; TGA: Therapeutic Goods Administration; ULN: upper limit of normal.

If you have any further recommendations or clarifications, please provide these in the following comment box:

COMMENT

Question 2: What are the reasonable **exclusion criteria for referral** for CAR-T therapy in patients with MM?

Summary of initial survey:

Consensus (≥5 out of 7 panellists) was reached on eight factors being considered as suitable exclusion criteria (see **green box** below).

Consensus was reached that renal dysfunction, prior treatment with allo-HSCT, and cardiovascular diseases are **not relevant** factors to be considered as part of the exclusion criteria.

No consensus was reached on **prior BCMA therapy**.

Summary of virtual meeting:

Panellists mostly agreed that **prior BCMA therapy** is **not relevant** for the exclusion criteria. One panellist mentioned that whether or not patients should be excluded based on prior BCMA therapy should align with MSAC guidelines.

Q2 statements with consensus (>70% or ⩾5/7 panellists):

- The following factors should be considered as appropriate exclusion criteria for referral:
    - Complex psychological issues (7/7)
    - Active or poorly controlled CNS disorder (including epilepsy, dementia or CNS involved autoimmune disorder)* (6/7)
    - Active HBV, HCV and/or other viral infection (e.g. EBV, CMV) (6/7)
    - Other active bacterial or fungal infection (e.g. tuberculosis) (5/7)
    - Live vaccines within 6 weeks of planned CAR-T infusion (5/7)
    - HIV (5/7)
    - Comorbidities conferring an expected life expectancy < 5 years (5/7)
    - Active uncontrolled graft-versus-host disease (5/7)

*In addition to the specific disorders included under this factor in the initial survey, one panellist further suggested pre-existing Parkinson's or movement disorder, history of seizures, or grade ≥2 peripheral neuropathy

Please review the results of the initial survey indicated by **blue columns** in the table below. In the same table, indicate your opinion on **agreement (Y/N)** for the non-consensus factors from the initial survey should be considered as part of the exclusion criteria for referral, as well as **importance (1, 2 or 3)** for each factor you believe should be considered.

You may keep the same answer you previously provided or change your answer in consideration of the outcomes of the initial survey and virtual meeting.

Please add a number from the scales presented in Table 1 to reflect the importance and practicality of each option:

Factor	Results of agreement in initial survey (number of panellists)	Agreement (Yes/No)	Results of importance in initial survey* (number of panellists)		Importance (Add number from 1 = 'very important' to 3 = 'not important')
			Important	Very important	
Active or poorly controlled CNS disorder (including epilepsy, dementia or CNS involved autoimmune disorder)	Consensus reached		1	4	1/2/3
Active infection with hepatitis virus (HBV, HCV) and/or others (e.g., EBV, CMV)	Consensus reached		0	4	1/2/3
Human immunodeficiency virus (HIV)	Consensus reached		0	4	1/2/3
Other active infection (active bacterial, fungal infection, active TB)	Consensus reached		1	3	1/2/3
Live vaccines within 6 weeks of planned CAR-T infusion	Consensus reached		1	1	1/2/3
Comorbidities conferring an expected life expectancy of < 5 years (e.g. secondary malignancies)	Consensus reached		1	2	1/2/3
Active uncontrolled graft-versus-host disease	Consensus reached		1	3	1/2/3
Complex psychological issues that may impact on compliance or patient safety	Consensus reached		1	2	1/2/3
Prior BCMA therapy	3/7	Y/N	0	0	1/2/3

**5/7 panellists provided scores for importance based on the scale defined in the first-round survey.*

Abbreviations: BCMA: B-cell Maturation Antigen; CAR-T: chimeric antigen receptor T; CMV: cytomegalovirus; CNS: central nervous system; EBV: Epsteine-Barr virus; HBV: hepatitis B virus; HCV: hepatitis C virus; HIV: human immunodeficiency virus; TB: tuberculosis.

If you have any further recommendations or clarifications, please provide these in the following comment box:

COMMENT

Question 3: What are the recommended elements considered for **prioritisation of referred patients**?

Summary of initial survey:

Consensus (≥5 out of 7 panellists) was reached on four factors (see **green box** below).

Consensus was also reached **on the following factors not being relevant**: i) time spent on the waiting list; ii) haematopoietic cell transplantation comorbidity index; iii) geographical location limitations; and iv) equity and equality considerations.

No consensus was reached on **disease refractoriness**, **age,** and **system/regional capacity**.

Summary of virtual meeting:

- Panellists mentioned the timing of CAR-T administration should be adapted regarding 'disease refractoriness'. For example, a patient with a fast serological relapse tendency should receive CAR-T before a patient with a slow relapse tendency. Panellists agreed to **rephrase** 'disease refractoriness' to '**bridgeability**'.
- One panellist suggested that frailty is more of concern in practice than **age**. Other panellists noted it is more of a consideration of eligibility rather than prioritisation. Overall, panellists **agreed that age is not an appropriate consideration for prioritisation of patients**, but instead would be a consideration for whether a patient is eligible or not.
- In practice, national capacity exists which means a patient can be moved between sites for treatment. Because of this, panellists agreed that '**system/regional capacity**' is **not a relevant factor when considering prioritisation**.

Q3 statements with consensus (>70% or ⩾5/7 panellists):

- Relevant patient-related factors as prioritisation criteria: disease burden (6/7), disease aggressiveness (6/7), likelihood of achieving clinical response (5/7).
- Relevant system/social-related factor as prioritisation criteria: availability of alternative treatment options (5/7).

Please review the results of the initial survey indicated by **blue columns** in the table below. In the same table, indicate your opinion on **agreement (Y/N)** for the non-consensus factors from the initial survey, as well as **importance and practicality (1, 2 or 3)** for each factor with which you agree.

You may keep the same answer you previously provided or change your answer in consideration of the outcomes of the initial survey and virtual meeting.

Please add a number from the scales presented in Table 1 to reflect the importance and practicality of each option:

**Factors**	**Results of agreement in initial survey** (number of panellists)	**Agreement (Yes/No)**	**Results of importance in initial survey*** (number of panellists)		**Importance** (Add number from 1 = 'very important' to 3 = 'not important')	**Results of ease of implementation in initial survey*** (number of panellists)		**Practicality** (Add number from 1 = 'very practical' to 3 = 'impractical')
			**Important**	**Very important**		**Practical**	**Very practical**	
**Patient-related factors:**								
Disease burden	Consensus reached		1	3	1/2/3	1	3	1/2/3
Disease aggressiveness	Consensus reached		1	3	1/2/3	1	1	1/2/3
Likelihood of achieving clinical response	Consensus reached		1	1	1/2/3	0	1	1/2/3
Age	3/7	Y/N	1	1	1/2/3	0	2	1/2/3
Bridgeability	Newly suggested factor during meeting	Y/N	NA		1/2/3	NA		1/2/3
Absence of active/uncontrolled infection	Newly suggested factor during meeting	Y/N	NA		1/2/3	NA		1/2/3

Factors	Results of agreement in initial survey (number of panellists)	Agreement (Yes/No)	Results of importance in initial survey* (number of panellists)		Importance (Add number from 1 = 'very important' to 3 = 'not important')	Results of ease of implementation in initial survey* (number of panellists)		Practicality (Add number from 1 = 'very practical' to 3 = 'impractical')
			Important	Very important		Practical	Very practical	
**System/social-related factors:**								
Availability of alternative treatment options	Consensus reached		2	0	1/2/3	1	1	1/2/3
System/regional capacity	4/7	Y/N	1	1	1/2/3	1	1	1/2/3
Access to appropriate social support (e.g. carer)	Newly suggested factor during meeting	Y/N	NA		1/2/3	NA		1/2/3

**5/7 panellists provided scores for importance and practicality based on the scale defined in the first-round survey.*

Abbreviations: NA: not applicable; Y/N: yes/no.

If you any further recommendations or clarifications, please provide these in the following comment box:

COMMENT

Question 4-A: What are the necessary **screening practices** for patient assessment at the CAR-T treatment centres, for patients who have been referred for CAR-T treatment?

Summary of initial survey:

Consensus (≥5 out of 7 panellists) was reached on four screening practices (**green box**).

Consensus was also reached that whole-body MRI, CT, or PET/CT are **not relevant** for screening (2/7).

No consensus was reached on conducting neurological investigations or a pregnancy test.

Summary of virtual meeting:

- The panellists mentioned a possible misinterpretation of 'neurological investigations' and suggested to **rephrase it as 'baseline neurocognitive assessment'.**
- Two panellists who did not select 'pregnancy test' explained that pregnancy tests are **only appropriate for patients who are of childbearing age. But if patients are of childbearing age, panellists agreed that this would be an appropriate** screening practice to include.
- One panellist added that **screening for infections** should be done prior to cell collection.

Q4 statements with consensus (>70% or ⩾5/7 panellists):

- Necessary screening practices include full blood count (6/7), full biochemistry (6/7), adequate respiratory function (6/7), and adequate cardiac function (6/7).

Note: testing for infectious diseases is mandated by the TGA, for which survey of consensus would be not necessary, therefore it is not listed in the question of agreement and criteria/cut-off values (but included in the question of assessment timing).

Please review the results of the initial survey indicated by **blue columns** in the table below. In the same table, indicate your opinion on **agreement (Y/N)** for the non-consensus factors from the initial survey.

You may keep the same answer you previously provided or change your answer in consideration of the outcomes of the initial survey and virtual meeting.

**Factors**	**Results of agreement in initial survey** (number of panellists)	**Agreement (Yes/No)**
Baseline neurocognitive assessment	Newly suggested factor during meeting (which has replaced 'neurological investigations' (4/7))	Y/N
Screening on infections	Newly suggested factor in first round	Y/N
Pregnancy test (if patients of childbearing age)	4/7	Y/N

If you have any further recommendations or clarifications, please provide these in the following comment box:

COMMENT

In the initial survey, panellists provided insights on **criteria/cut-off values** for screening factors. Please review the following statements and **indicate those with which you agree (Y/N)**.

Please note, if in the **previous table you have not selected that you agree** with the factors being part of the screening practice, **please do not provide an answer for this factor in the table below.**

You may keep the same answer you previously provided or change your answer in consideration of the outcomes of the initial survey and virtual meeting.

**Factors***	**Results of agreement in initial survey** (number of panellists)		**Agreement (Yes/No)**
Full Blood count	Haemoglobin (3/6)	Nil (2/3)	Y/N
		≥80g/L (1/3)	Y/N
	Platelets (4/6)	$>50\times10^9$/L (4/6)	Y/N
	Lymphocytes (5/6)	$>0.5\times10^9$/L (2/5)	Y/N
		$>0.3\times10^9$/L (1/5)	Y/N
		Nil (1/5)	Y/N
Full biochemistry	Corrected serum calcium (3/6)	<3 mmol/L (1/3)	Y/N
		ULN (2/3)	Y/N
Screening on infections†	QuantiFERON gold (newly suggested by one panellist in initial survey)	NA	Y/N

Note:
**Criteria/cut-off values of some factors are also discussed in Q1, including cardiac function, respiratory function, liver function renal function, and neurocognitive assessment (MRI, LP, CSF). To avoid duplication, those factors are not included in this table.*
†The panellist also suggested HBV, HCV and HIV under factor of "screening on infections", which are also covered in Q1, therefore not included in this table to avoid duplication.

If you have any further recommendations or clarifications, please provide these in the following comment box:

COMMENT

Question 4-B: Timing of assessment based on Q1 and Q4-A.

In the initial survey, panellists provided insights on **assessment timing** (some factors are from Q1, for which panellists provided timings other than at referral, and therefore are integrated in this question).

Please review the following statements and **indicate those with which you agree (Y/N)**.

For the factors that you **have previously stated should not be considered at all (see Q1 and Q4-A)**, please **do not provide an answer for this factor in the table below**.

You may keep the same answer you previously provided or change your answer in consideration of the outcomes of the initial survey and virtual meeting.

Factors	Assessment timing suggested in the initial survey* (number of panellists)	Agreement (Yes/No), please leave the cells blank if you do not believe the factor should be included
Adequate cardiac function	Pre-apheresis/pre-collection (3/7)	Y/N
	At screening (1/7)	Y/N
	Pre- lymphodepletion (1/7)	Y/N
Adequate respiratory function	At screening (1/6)	Y/N
	Pre-apheresis/pre-collection (1/6)	Y/N
	Pre-lymphodepletion (1/6)	Y/N
	Post-lymphodepletion (1/6)	Y/N
	At infusion (1/6)	Y/N
Adequate renal function	At screening (1/6)	Y/N
	Pre-apheresis/pre-collection (1/5)	Y/N
	Pre-lymphodepletion (2/5)	Y/N
	At lymphodepletion (1/6)	Y/N
	Post-lymphodepletion (1/5)	Y/N
	At infusion (1/5)	Y/N
Adequate liver function	Pre-apheresis/pre-collection (1/5)	Y/N
	Pre-lymphodepletion (1/5)	Y/N
	Post lymphodepletion (1/5)	Y/N
	At infusion (1/5)	Y/N
Expected life expectancy	Pre-apheresis/pre-collection (2/6)	Y/N
	At infusion (1/6)	Y/N
Frailty score	Pre-apheresis/pre-collection (2/5)	Y/N
	Pre-lymphodepletion (1/5)	Y/N

Factors	Assessment timing suggested in the initial survey* (number of panellists)	Agreement (Yes/No), please leave the cells blank if you do not believe the factor should be included
	At infusion (1/5)	Y/N
Absence of active/uncontrolled infection (Screening on infection)	Pre-apheresis/pre-collection (2/4)	Y/N
	At lymphodepletion (1/4)	Y/N
	At infusion (2/4)	Y/N
ECOG score	Pre-lymphodepletion (1/7)	Y/N
	At infusion (1/7)	Y/N
Pace of disease progression	Post referral (1/6)	Y/N
	At infusion (1/6)	Y/N
Neurocognitive assessment	Post referral (1/4)	Y/N
	At baseline (1/4)	Y/N
	At infusion (1/4)	Y/N
Pregnancy test	At infusion (1/4)	Y/N
	Monthly for WOCBP during treatment (1/4)	Y/N
Full blood count	At referral (1/6)	Y/N
	At infusion (1/6)	Y/N
	Platelets: Pre-infusion and lymphocytes: pre-collection (1/6)	Y/N

**For factors from Q1(referral criteria), it is assumed that the ones reached consensus of agreement should be assessed before referral, therefore not included in this table.*
Abbreviations: ECOG: Eastern Cooperative Oncology Group, WOCBP: Women of child-bearing potential.

If you have any further recommendations or clarifications, please provide these in the following comment box:

COMMENT

Part 2: Pre-CAR-T management and infusion

Question 5: What are the recommendations on wash-out practices prior to leukapheresis?

Summary of initial survey:

Consensus (⩾5 out of 7 panellists) was reached on seven agents which need a wash-out before leukapheresis (**green box**).

No consensus was reached on a wash-out period for CNS radiotherapy.

Summary of virtual meeting:

- Standard lymphoma guidelines recommend general radiotherapy. The panellists explained a wash-out period is required for radiotherapy in general and not only for CNS radiotherapy. Therefore 'CNS radiotherapy' will be replaced by '**radiotherapy**' in the proposed practices.
- Panellists stressed the necessity of a wash-out period for **bendamustine** and suggested a minimal timeframe of 24 weeks.

Q5 agents with consensus (>70% or ⩾5/7 panellists):

- The following procedures/agents need a wash-out before leukapheresis: allo-HSCT (7/7), systemic corticosteroids (6/7), proteasome inhibitors (6/7), immunomodulatory drugs (6/7), high-dose chemotherapy (6/7), donor lymphocyte infusion (5/7), and anti-CD38 monoclonal antibodies (5/7).

Please review the results of the initial survey indicated by **blue columns** in the table below. In the same table, indicate your opinion on **agreement (Y/N)** for the non-consensus factors from the initial survey, as well as **importance (1, 2 or 3)** for each factor with which you agree.

You may keep the same answer you previously provided or change your answer in consideration of the outcomes of the initial survey and virtual meeting.

Please add a number from the scales presented in Table 1 to reflect the importance of each option:

Factors	Results of agreement in initial survey (number of panellists)	Agreement (Yes/No)	Results of importance in initial survey* (number of panellists)		Importance (Add number from 1 = 'very important' to 3 = 'not important')
			Important	Very important	
Allo-HSCT (off immunosuppression and GvHD free)	Consensus reached		2	1	1/2/3
Donor lymphocyte infusion	Consensus reached		1	1	1/2/3
High-dose chemotherapy	Consensus reached		3	0	1/2/3
Systemic corticosteroids	Consensus reached		1	2	1/2/3
Proteasome inhibitors	Consensus reached		2	1	1/2/3
Immunomodulatory drugs	Consensus reached		2	0	1/2/3
Anti-CD38 monoclonal antibodies	Consensus reached		2	0	1/2/3
Radiotherapy	Newly suggested factor during virtual meeting	Y/N	NA		1/2/3
Bendamustine	Newly suggested factor by one panellist in initial survey	Y/N	0	1	1/2/3

**Only 4/7 panellists provided scores for importance based on the scale defined in the first-round survey*

Abbreviations: allo-HSCT: allogeneic hematopoietic stem cell transplantation; GvHD graft-versus-host disease; NA: not applicable.

If you have any further recommendations or clarifications, please provide these in the following comment box:

COMMENT

In the initial survey, panellists provided insights on the duration that should be allowed for wash-out.

Please review the following statements and **indicate those with which you agree (Y/N)**. For the factors that you **<u>have stated in the previous table should not be considered</u>**, please **<u>do not provide an answer for this factor in the table below</u>**.

You may keep the same answer you previously provided or change your answer in consideration of the outcomes of the initial survey and virtual meeting.

**Factor**	**Minimal wash-out timeframe** in the initial survey (Number of panellist)	**Agreement (Yes/No), please leave the cells blank if you have previously stated the corresponding factor should not be included**
Allo-HSCT (off immunosuppression and GvHD free)	24 weeks (1/7)	Y/N
	12 weeks (1/7)	Y/N
	8 weeks (2/7)	Y/N
High-dose chemotherapy	8 weeks (2/6)	Y/N
	4 weeks (2/6)	Y/N
	2 weeks (2/6)	Y/N
Immunomodulatory drugs	4 weeks (1/6)	Y/N
	2 weeks (3/6)	Y/N
	7 days (2/6)	Y/N
Proteasome inhibitors	2 weeks (4/6)	Y/N
	7 days (2/6)	Y/N
Systemic corticosteroids	2 weeks (4/6)	Y/N
	3 to 7 days (2/6)	Y/N
Anti-CD38 monoclonal antibodies	4 weeks (2/5)	Y/N
	2 weeks (3/5)	Y/N
Donor lymphocyte infusion	8 weeks (3/5)	Y/N
	4 weeks (2/5)	Y/N
CNS radiotherapy	4 weeks (2/4)	Y/N
	2 weeks (1/4)	Y/N
Bendamustine	12 weeks (Newly suggested factor in initial survey)	Y/N
	24 weeks (Newly suggested factor in virtual meeting)	Y/N
Radiotherapy	4 weeks (Newly suggested factor in initial survey)	Y/N
	2 weeks (Newly suggested factor in initial survey)	Y/N

Abbreviations: Allo-HSCT: allogeneic hematopoietic stem cell transplantation; CNS: central nervous system; GvHD graft-versus-host disease.

If you have any further recommendations or clarifications, please provide these in the following comment box:

COMMENT

Question 6-A: What are the recommendations on optimal practices for bridging therapy before initiating lymphodepletion?

Summary of initial survey:

Consensus (≥5 out of 7 panellists) was reached on three statements (see **green box** for details)

No consensus was reached on the following statement: specific interventions should be avoided as bridging therapy.

Summary of virtual meeting:

- The panellists agreed that it is not appropriate to be prescriptive on which interventions should be avoided, given the variability of patients' exposure to agents and the progression speed of the disease. Therefore, **it is not necessary to avoid specific interventions.**

Q6-A statements with consensus (>70% or ⩾5/7 panellists):

- Bridging therapy may not be necessary or beneficial in some circumstances (6/7)
- Therapies that patients are naïve to can be used in bridging therapy (6/7)
- Washout periods for bridging therapy are necessary (7/7)

Please review the statement from the initial survey in which consensus was not reached, indicated by the **blue column** in the table below. In the same table, indicate your opinion on **agreement with the statement (Y/N)**.

You may keep the same answer you previously provided or change your answer in consideration of the outcomes of the initial survey and virtual meeting.

Questions	**Results of agreement in initial survey** (number of panellists)	Agreement
Are there any interventions that should be avoided as bridging therapy?	Yes (4/7)<br>No (3/7)	☐ Yes<br>☐ No

If you have any further recommendations or clarifications, please provide these in the following comment box:

COMMENT

Question 6-B: What are the **factors** considered when deciding the **type and duration of bridging therapy**?

Summary of initial survey:

Consensus (⩾5 out of 7 panellists) was reached on five factors to consider for bridging (see **green box** for details)

No consensus was reached on the disease refractoriness, the availability on the PBS, the prior lines of therapy used, or age.

Summary of virtual meeting:

- Panellists agreed to rephrase 'disease refractoriness' to '**bridgeability**' to avoid misinterpretation.
- In terms of '**availability on the PBS**', some panellists mentioned it was a crucial factor that impacts the decision. Others pointed out efficacy and general availability are factors to consider, not how the agent is accessed. Panellists in general agreed to apply broader phrasing here, specifically **'availability of the intervention'**.
- One panellist suggested '**prior lines of therapy**' should be considered among other criteria for the choice of bridging therapy.
- Age is **not relevant,** and one panellist suggested it should not be a criterion in the guidelines for bridging therapy.

Q6-B statements with consensus (>70% or ⩾5/7 panellists):

- The following factors are to be considered for bridging therapy: disease burden (6/7), disease aggressiveness (6/7), response to prior lines of therapy (5/7), patient's likelihood to tolerate bridging therapy (5/7), and historical CAR-T manufacturing time (5/7).

Please review the results of the initial survey indicated by **blue columns** in the table below. In the same table, indicate your opinion on **agreement (Y/N)** for the non-consensus factors from the initial survey, as well as **importance and practicality (1, 2, 3)** for each factor with which you agree.

You may keep the same answer you previously provided or change your answer in consideration of the outcomes of the initial survey and virtual meeting.

Please add a number from the scales presented in Table 1 to reflect the importance and practicality of each option:

Factors	Results of agreement in initial survey (number of panellists)	Agreement (Yes/No)	Results of importance in initial survey* (number of panellists selected important/very important)		Importance (Add number from 1 = 'very important' to 3 = 'not important')	Results of practicality in initial survey* (number of panellists selected practical/very practical)		Practicality (Add number from 1= 'very practical' to 3= 'impractical')
			Important	Very important		Practical	Very practical	
Disease aggressiveness	Consensus reached		0	3	1/2/3	0	1	1/2/3
Disease burden	Consensus reached		0	3	1/2/3	0	2	1/2/3
Historical CAR-T manufacturing time	Consensus reached		0	2	1/2/3	0	2	1/2/3
Response to prior lines of therapy	Consensus reached		1	2	1/2/3	1	1	1/2/3
Patient's likelihood to tolerate bridging therapy	Consensus reached		0	3	1/2/3	1	1	1/2/3
Prior lines of therapy used	3/7	Y/N	1	1	1/2/3	1	1	1/2/3

**Factors**	**Results of agreement in initial survey** (number of panellists)	**Agreement (Yes/No)**	**Results of importance in initial survey*** (number of panellists selected important/very important)		**Importance** (Add number from 1 = 'very important' to 3 = 'not important')	**Results of practicality in initial survey*** (number of panellists selected practical/very practical)		**Practicality** (Add number from 1= 'very practical' to 3= 'impractical')
			**Important**	**Very important**		**Practical**	**Very practical**	
Availability of the intervention	Newly suggested during meeting (to replace 'availability on the PBS' (4/7))	Y/N	NA		1/2/3	NA		1/2/3
Age	3/7	Y/N	1	1	1/2/3	0	1	1/2/3
Bridgeability	Newly suggested factor during meeting	Y/N	NA		1/2/3	NA		1/2/3

**Only 3/7 panellists provided scores for importance and practicality based on the scale defined in the first-round survey.*

Abbreviations: CAR-T: chimeric antigen receptor T; PBS: pharmaceutical benefit scheme; NA: not applicable.

If you have any further recommendations or clarifications, please provide these in the following comment box:

COMMENT

Question 7: What are the recommendations on the necessary and appropriate **practices for lymphodepletion**?

Summary of initial survey:

Consensus (≥5 out of 7 panellists) was reached on three statements (see **green box** for details)

No consensus was reached on the following statement: 'consultation with a neurologist for patients with experience or risk of ICANS'.

Summary of virtual meeting:

- In practice, it is a challenge to provide **neurologist consultation** prior to CAR-T therapy for some states in Australia, because there is lack of dedicated resources in CAR-T therapy centres. Although neurologist consultation is a best practice, it has an 'access and availability' issue. It would therefore be practical to rephrase the statement to indicate neurologist consultation required if available.

Q7 statements with consensus (>70% or ⩾5/7 panellists):

- Lymphodeplete patients with the combination fludarabine / cyclophosphamide for 3 days (6/7).
- General doses for lymphodepletion are 25-30 mg/m^2 for fludarabine and 250-300 mg/m^2 for cyclophosphamide (6/7).
- Dose adjustment for fludarabine based on creatinine clearance (5/7).

Please review the results of the non-consensus statement in the initial survey in the **blue column** and indicate your opinion in the table below.

You may keep the same answer you previously provided or change your answer in consideration of the outcomes of the initial survey and virtual meeting.

Statement	**Results of agreement in initial survey** (number of panellists)	**Agreement (Yes/No)**
Consultation with a neurologist for patients with experience or risk of ICANS is the optimal practice if resources are available to do so[3].	4/7	Y/N

Abbreviation: ICANS: immune effector cell-associated neurotoxicity syndrome.

[3] The statement has been updated from the first round based on panellists' recommendation during meeting.

If you have any further recommendations or clarifications, please provide these in the following comment box:

COMMENT

Question 8: What are the recommendations on the **best practices for CAR-T infusion?**

Summary of initial survey:

Consensus (≥5 out of 7 panellists) was reached on two statements (see **green box** for details)

No consensus reached on the following statements:

- 'Necessity to wait for a specific number of days between lymphodepletion and infusion initiation'.
- 'Necessity to avoid the use of corticosteroids during infusion'.
- 'Hospitalisation post CAR-T is a must'

Summary of virtual meeting:

- All panellists agreed on a minimum of 48h between lymphodepletion and infusion initiation.
- Regarding the use of corticosteroids, panellists agreed that it was better not to use but **not 'necessary to avoid'** their use, and therefore would agree answer is "no" to this question.
- Outpatient models depend on hospital availability and patient profile. The panellists agreed that **hospitalisation** post CAR-T was **not a must**.

Q8 statements with consensus (>70% or ⩾5/7 panellists):

- The use of paracetamol and antihistamine products to minimize infusion reaction is appropriate (5/7).
- CAR-T products that are out of specification can be used (5/7).

Please review the results of the initial survey indicated by **blue columns** in the table below. In the same table, indicate your opinion on **agreement (Y/N)** for the non-consensus factors from the initial survey.

You may keep the same answer you previously provided or change your answer in consideration of the outcomes of the initial survey and virtual meeting.

Statement	**Results of agreement in initial survey** (number of panellists)	Agreement (Yes/No)
**Efficacy related practices**:		

Statement	Results of agreement in initial survey (number of panellists)	Agreement (Yes/No)
Is it necessary to wait for 48h after lymphodepletion before infusion initiated?	Yes (4/7)<br>No (2/7)<br>Uncertain (1/7)	☐ Yes<br>☐ No
**Safety related practices**:		
Is it necessary to avoid using corticosteroids during infusion?	Yes (3/7)<br>No (4/7)	☐ Yes<br>☐ No
Is hospitalisation post CAR-T a must?	Yes (4/7)<br>No (3/7)	☐ Yes<br>☐ No

Abbreviation: CAR-T: chimeric antigen receptor T.

If you have any further recommendations or clarifications, please provide these in the following comment box:

COMMENT

Part 3: Post CAR-T management

Question 9: What are the necessary practices after CAR-T therapy infusion?

Summary of initial survey:

Consensus (⩾5 out of 7 panellists) was reached on one factor associated with MNT risk, four practices linked to infection monitoring, and three evaluation criteria for CAR-T therapy (see **green box** for details).

Consensus was also reached that assessment of CAR-T expansion and persistence, and assessment of absolute lymphocyte count are **not relevant** factors associated with MNT risk.

No consensus was reached on the presence of grade 2 or higher CRS as a factor associated with MNT risk. Additionally, consensus was not reached for organ function as an evaluation factor after CAR-T infusion.

Summary of virtual meeting:

- Panellists highlighted the **absence of a causal relationship** between **grade 2 CRS and MNT risk**, and mentioned that the only existing causal relationship is with hyperexpansion of CAR-T cells.
- Panellists pointed out that the term '**organ function**' is too broad and suggested to rephrase with '**biochemical assessments**' which would be routinely done in contrast more extensive assessments such as echocardiogram.
- One panellist suggested to add a neurocognitive assessment prior to CAR-T infusion to compare it with the cognitive functions of the patients after CAR-T therapy. Panellists agreed with the approach and neurological assessment before and after CAR-T infusion was added to this survey.

Q9 Statements with consensus (>70% or ⩾5/7 panellists):

- Factors associated with MNT risk: the assessment of tumour burden at baseline (5/7).
- Management of infection: use of intravenous immunoglobulin therapy (6/7), use of growth factor support (5/7), revaccination (5/7), and prophylactic antimicrobial interventions (5/7).
- Evaluation criteria: ASTCT grading scale for CRS (7/7), ASTCT grading scale for ICANS (7/7), and CTCAE for infectious condition (7/7).

Please review the results of the initial survey indicated by **blue columns** in the table below. In the same table, indicate whether you agree or disagree (Y/N) with the inclusion of each factor, practice, or criterion.

You may keep the same answer you previously provided or change your answer in consideration of the outcomes of the initial survey and virtual meeting.

Factors/Practices/Criteria	**Results of agreement in initial survey** (number of panellists)	Agreement (Yes/No)
**Factors associated with MNT risk**:		
Presence of grade 2 or higher CRS	4/7	Y/N
Assessment of CAR-T expansion and persistence	1/7 during first round, but multiple panellists mentioned it as a factor during meeting	Y/N
Neurocognitive assessment before and after CAR-T infusion	Newly suggested factor by one panellist in the first round	Y/N
**Evaluation Criteria**		
Organ function	3/7	Y/N
Biochemical assessments	Newly suggested factor during meeting	Y/N

Abbreviations: CAR-T: chimeric antigen receptor T; CRS: cytokine release syndrome; MNT: movement and neurocognitive treatment.

If you have any further recommendations or clarifications, please provide these in the following comment box:

COMMENT

Question 10: What are the recommendations on **monitoring schemes** for adverse events and disease progression associated with subsequent anti-myeloma therapy?

Summary of initial survey:

Consensus (⩾5 out of 7 panellists) was reached for 4 recommendations on acute toxicity monitoring, 3 recommendations on chronic toxicity monitoring, and 4 elements of clinical response (see **green box** for details).

Consensus was reached that MRI for chronic toxicity monitoring and clinical response evaluation are **not relevant**.

No consensus was reached for the chronic toxicity monitoring of delayed tumour lysis syndrome/CRS/ICANS on the evaluation of clinical response with serum immunofixation, PET-CT, CAR-T monitoring, and bone marrow cytology.

Summary of virtual meeting:

- Panellists suggested to specify the definition of acute and chronic monitoring with the following timeframe: acute = within 3 months post-infusion vs. chronic = from 3 months post-infusion.

- Panellists highlighted that in practice, CAR-T monitoring is usually **not possible**. (Note: This factor is rephrased in 'CAR-T persistence monitoring' for clarity.)
- One panellist mentioned being cautious on recommending the use of PET-CT because it is not funded in Australia. Panellists agreed that it could be applied where appropriate but should **not be mandatory**.
- The panellists suggested to rephrase 'bone marrow cytology' to 'bone marrow assessment'.

Q10 Statements with consensus (>70% or ⩾5/7 panellists):

- Monitoring factors for acute toxicity (within 3 months after CAR-T infusion): infections (7/7), ICANS (7/7), CRS (7/7), and full blood count (6/7).
- Monitoring factors for chronic toxicity (from 3 months after CAR-T infusion): neurological status (7/7), infections (7/7), and full blood count (5/7).
- Clinical response evaluation: serum M quantification (7/7), serum free light chain (7/7), standard follow-up (6/7), and minimal residual (MRD) status (5/7).

Please review the results of the initial survey indicated by **blue columns** in the table below. In the same table, indicate whether you agree or disagree (Y/N) with the inclusion of each test or parameter.

You may keep the same answer you previously provided or change your answer in consideration of the outcomes of the initial survey and virtual meeting.

**Test/Parameter**	**Results of agreement in initial survey** (number of panellists)	**Agreement (Yes/No)**
**Acute toxicity monitoring (within 3 months post CAR-T infusion):**		
MoCA	Newly suggested test during meeting	Y/N
**Chronic toxicity monitoring (from 3 months post CAR-T infusion)**:		
Delayed tumour lysis syndrome/CRS/ICANS	4/7	Y/N
**Clinical response**		
Serum immunofixation	4/7	Y/N
PET-CT	4/7	Y/N
CAR-T persistence monitoring	3/7	Y/N

Test/Parameter	**Results of agreement in initial survey** (number of panellists)	Agreement (Yes/No)
Bone marrow assessment	3/7	Y/N

Abbreviations: CRS: cytokine release syndrome; ICANS: immune effector cell-associated neurotoxicity syndrome; MoCA: Montreal cognitive assessment; PET-CT: positron emission tomography-computed tomography.

If you have any further recommendations or clarifications, please provide these in the following comment box:

COMMENT

In the initial survey, panellists provided insights on **practice strategies** for post-CAR-T monitoring. Please review the following statements and **indicate those with which you agree**.

Please note, if in the **previous table you have not selected that you agree** with the factors being part of monitoring, **please do not provide an answer for this factor in the table below.**

All strategies listed in the table were suggested by panellists, **if you disagree with the available suggestions, you can provide a new strategy in the 'New suggested strategy' column** for each factor.

Factors	Strategies suggested by panellists in the initial survey	**Agreement (Yes/No)**, please leave the cells blank if you do not agree with a factor at all	**New suggested strategy** Please enter your suggested strategy in the comment box
**Acute toxicity phase (within 3 months post CAR-T infusion):**			
CRS	Daily during first 14 days when hospitalised, then twice per week post discharge for first month, then every 4 weeks until 3 months	Y/N	COMMENT
Full blood count	Daily during first 14 days when hospitalised, then twice per week post discharge for first month, then every 4 weeks until 3 months	Y/N	COMMENT
	Every visit for outpatient, or daily for inpatient	Y/N	
	Every week	Y/N	

**Factors**	**Strategies suggested by panellists in the initial survey**	**Agreement (Yes/No)**, please leave the cells blank if you do not agree with a factor at all	**New suggested strategy** Please enter your suggested strategy in the comment box
ICANS	Every day when hospitalised, then twice per week for the first month, then weekly for the second month	Y/N	COMMENT
	Twice daily during 14 days when hospitalised, then daily post discharge for first month, then twice per week.	Y/N	
Infections	Every day for 3 months	Y/N	COMMENT
	Every day for 14 days (strategy after 14 days not provided)	Y/N	
	Every week for 3 months	Y/N	
	Every visit for 3 months	Y/N	
MNTs, cognitive status (MoCA)	Daily during first 14 days (inpatient), then twice per week post discharge for the first month, and then every 4 weeks for second and third 3 months.	Y/N	COMMENT
**Chronic toxicity monitoring (from 3 months post CAR-T infusion):**			
Full blood count	Minimally every 3 months	Y/N	COMMENT
	Every month for 1 year	Y/N	
Infections	Every month for 1 year	Y/N	COMMENT
	Every month for 5 years	Y/N	
Neurological status	1 year (frequency not indicated); post 1 year: ongoing if clinically indicated	Y/N	COMMENT
	Every month for 1 year	Y/N	
	Every month for 5 years	Y/N	
	Every visit (timeframe not indicated)	Y/N	
Delayed tumour lysis syndrome/CRS/ICANS	Every review for 1 year	Y/N	COMMENT
	Every month for 1 year	Y/N	

Factors	Strategies suggested by panellists in the initial survey	Agreement (Yes/No), please leave the cells blank if you do not agree with a factor at all	New suggested strategy Please enter your suggested strategy in the comment box
MNTs, cognition	Every 3 months (timeframe not indicated)	Y/N	COMMENT
**Clinical response:**			
Serum free light chain	Monthly for first 6 months then every 3 months for lifetime	Y/N	COMMENT
	Lifetime, frequency depends on progress	Y/N	
	Every month for 5 years	Y/N	
	Every month for lifetime	Y/N	
Serum M protein quantificatio n	Lifetime, frequency depends on progress	Y/N	COMMENT
	Every month for 5 years	Y/N	
	Every month for lifetime	Y/N	
Standard follow-up	Twice per week post discharge until first month, then every 4 weeks until 3 months, then every 3 months for lifetime	Y/N	COMMENT
	Depends upon time post Rx and variable from monthly to 3 monthly (timeframe lifetime)	Y/N	
	Every month for 5 years	Y/N	
	Every 3 months for lifetime	Y/N	
Minimal residual disease (MRD) status	Every 6 months for lifetime	Y/N	COMMENT
	Every 3 months for 15 years	Y/N	
	Every year for 5 years	Y/N	
PET-CT	As clinically indicated	Y/N	COMMENT
	Every year for 5 years	Y/N	
Serum immunofixati on	Every month for 5 years	Y/N	COMMENT
	Every month for lifetime	Y/N	
	Every 3 months	Y/N	

**Factors**	**Strategies suggested by panellists in the initial survey**	**Agreement (Yes/No)**, please leave the cells blank if you do not agree with a factor at all	**New suggested strategy** Please enter your suggested strategy in the comment box
Bone marrow assessment	Lifetime, frequency depends on progress	Y/N	COMMENT
	Upon indication, unexplained cytopenia, suspicion of secondary BM malignancies, progressive disease	Y/N	
CAR-T persistence monitoring	Every month for 5 years	Y/N	COMMENT
	Every month for lifetime	Y/N	

Abbreviations: BM: bone marrow; CAR-T: chimeric antigen receptor T; CR: complete response; CRS: cytokine release syndrome; ICANS: immune effector cell-associated neurotoxicity syndrome; MNTs: movement and neurocognitive treatment; MoCA: Montreal cognitive assessment; MRD: minimal residual disease; PET-CT: positron emission tomography-computed tomography; Rx: treatment regimen.

If you have any further recommendations or clarifications, please provide these in the following comment box:

COMMENT
