# Supplementary material for: Management of CAR-T cell therapy in patients with multiple myeloma: a systematic review and expert consensus in Australia
Source: Front Oncol. 2025 Jan 21;14:1535869. doi: 10.3389/fonc.2024.1535869 (PMC11790593; doi:10.3389/fonc.2024.1535869)
Supplement: Supplementary file 3 [file DataSheet3.docx]

Supplementary Material I

# Methods of Systematic Literature Review

The SLR search strategy was designed to identify evidence on CAR-T cell therapy implementation for patients with MM, including patient identification, referral, screening, selection and prioritization, bridging therapy, infusion, and post-infusion management. EMBASE, MEDLINE, and MEDLINE In-Process were searched from inception to March 28, 2023, using subject headings (Emtree and MeSH) and free text terms, to address each aspect of the research question (Tables S1 and S2). Hand searches of clinical trial registries and conference proceedings from January 1, 2020 to April 28, 2023 were conducted to capture data from unpublished studies. Patient association websites and Google Scholar were searched to identify relevant reports published by patient registries and associations, or governments. The resources for hand search included:

Conference proceedings:

- European Myeloma Network Meeting (www.myeloma-europe.org/)
- International Myeloma Society Annual Meeting (www.myelomasociety.org/)
- Lymphoma and Myeloma Congress (www.lymphomaandmyeloma.oncnet.com/)
- The European hematology association (EHA) (www.ehaweb.org/)
- The American society of clinical oncology (ASCO) (www.asco.org/)
- The American society of hematology (ASH) (www.hematology.org/)
- Controversies in multiple myeloma (COMy) (www.comylive.cme-congresses.com/)

Clinical trials registries:

- ClinicalTrials.gov
- EU Clinical Trials Register (EUCTR) (www.clinicaltrialsregister.eu/)

Patient association websites:

- Myeloma Australia (www.myeloma.org.au/)
- Lymphoma Australia (www.lymphoma.org.au/)

Google scholar:

- A hand search of grey literature was conducted using Google Scholar to identify relevant reports published by patient registries, patient associations, or governments

The results of all searches were combined into a single reference library and duplicates were removed using Zotero (Corporation for Digital Scholarship).

The population, intervention, comparator, outcomes, and study type (PICOS) framework was used to define prespecified study inclusion criteria (Table S3) (1). Observational or interventional studies that comprised patients with MM treated with CAR-T cell therapy were included, together with clinical guidelines, expert consensus reports, and advisory board publications to identify outcomes related to diagnosis and patient management. Reviews by authors at Australian institutions were included for cross-referencing purposes. There were no restrictions on geography or language.

The study selection process was documented in accordance with the preferred reporting items for systematic reviews and meta-analyses (PRISMA) statement 2020 (2). Initially, titles and abstracts were screened against the prespecified inclusion criteria. Complete texts of titles and abstracts deemed potentially relevant were then obtained and reviewed in full according to the same inclusion criteria. Screening was performed by two independent reviewers in parallel with differences resolved by a third reviewer. A reviewer not involved in the initial screening performed a quality check on 20% of the screened articles. Two independent reviewers extracted publication details, study characteristics, patient characteristics, and outcomes from the included studies into a tabular summary file.

Table S1. Search terms for EMBASE via [www.embase.com](http://www.embase.com) (date of the search: March 28, 2023)

| No. | Query | Results |
| --- | --- | --- |
| 1 | 'multiple myeloma'/exp OR 'multiple myeloma*' OR myeloma OR (myeloma* NEAR/1 multiple*) | 136419 |
| 2 | 'myeloma*, plasma-cell' OR 'plasma-cell myeloma*' OR 'plasma cell myeloma' OR 'cell myeloma*, plasma' OR 'myeloma*, plasma cell' OR 'plasma cell myeloma*' OR 'myeloma-multiple' OR 'myeloma* multiple*' | 3270 |
| 3 | (kahler NEAR/1 disease) OR myelomatos?s | 877 |
| 4 | #1 OR #2 OR #3 | 136518 |
| 5 | ('antigen receptor*' NEAR/1 chimeric) OR ('chimeric antigen' NEAR/1 receptor*) OR (chimeric NEAR/1 (immunoreceptor* OR 't-cell receptor*' OR 't-cell receptor*')) OR 'artificial t cell receptor*' OR 'artificial t-cell receptor*' OR (receptor* NEAR/1 'chimeric t-cell*') OR 'car-t' OR 'car-t cell*' | 26764 |
| 6 | 'bb 2121' OR 'ciltacabtagene autoleucel'/de OR 'idecabtagene vicleucel'/de | 523 |
| 7 | 'abecma'/de OR 'jnj 68284528' OR 'lcar-b38m car-t cells' OR 'carvykti'/de OR 'anti-bcma car t cell' OR 'ide-cel' | 624 |
| 8 | #5 OR #6 OR #7 | 26816 |
| 9 | 'clinical trial'/de | 1071138 |
| 10 | 'randomized controlled trial'/de | 757461 |
| 11 | 'controlled clinical trial'/de | 439480 |
| 12 | 'multicenter study'/de | 344431 |
| 13 | 'phase 1 clinical trial'/de OR 'phase 2 clinical trial'/de OR 'phase 3 clinical trial'/de OR 'phase 4 clinical trial'/de | 207050 |
| 14 | 'randomization'/exp | 95998 |
| 15 | 'single blind procedure'/de OR 'double blind procedure'/de | 248789 |
| 16 | 'crossover procedure'/de | 72372 |
| 17 | 'placebo'/de | 397211 |
| 18 | 'randomi?ed controlled trial$':ti,ab | 301757 |
| 19 | rct:ti,ab OR 'non rct':ti,ab | 50440 |
| 20 | (random$ NEAR/2 allocat$):ti,ab | 3 |
| 21 | (((treble OR triple) NEAR/1 blind$):ti,ab) OR 'single blind$':ti,ab OR 'double blind$':ti,ab | 239074 |
| 22 | placebo$:ti,ab | 353301 |
| 23 | #9 OR #10 OR #11 OR #12 OR #13 OR #14 OR #15 OR #16 OR #17 OR #18 OR #19 OR #20 OR #21 OR #22 | 2249934 |
| 24 | ('cross sectional' NEAR/1 (study OR studies)):ti,ab | 313071 |
| 25 | (epidemiologic$ NEAR/1 (study OR studies)):ti,ab | 35274 |
| 26 | (observational NEAR/1 (study OR studies)):ti,ab | 233305 |
| 27 | ('follow up' NEAR/1 (study OR studies)):ti,ab | 77034 |
| 28 | ('case control' NEAR/1 (study OR studies)):ti,ab | 160746 |
| 29 | (cohort NEAR/1 (study OR studies)):ti,ab,kw | 459835 |
| 30 | 'cohort analysis'/de | 931252 |
| 31 | 'prospective study'/de | 816769 |
| 32 | 'retrospective study'/de | 1355277 |
| 33 | 'longitudinal study'/de | 183020 |
| 34 | 'family study'/de | 26246 |
| 35 | 'case control study' | 245309 |
| 36 | 'clinical study'/de | 161344 |
| 37 | 'register'/exp OR registr*:ti,ab | 500066 |
| 38 | real NEAR/3 world | 121228 |
| 39 | ('real world' OR 'real world') NEAR/2 (data OR evidence OR research OR study OR studies OR trial*) | 38579 |
| 40 | hospital NEAR/3 record* | 32059 |
| 41 | claim* NEAR/3 (medical OR database*) | 22398 |
| 42 | #24 OR #25 OR #26 OR #27 OR #28 OR #29 OR #30 OR #31 OR #32 OR #33 OR #34 OR #35 OR #36 OR #37 OR #38 OR #39 OR #40 OR #41 | 4162598 |
| 43 | 'clinical pathway'/exp OR 'clinical protocol'/exp OR 'consensus'/exp OR 'guideline*' OR 'practice guideline*' OR 'clinical decision rule*' | 1140299 |
| 44 | 'position statement*':ti,ab,kw OR 'practice parameter*':ti,ab,kw OR 'policy statement*':ti,ab,kw OR 'best practice*':ti,ab,kw OR (((practice OR treatment* OR clinical) NEAR/2 guideline*):ab) | 152544 |
| 45 | 'patient* selection' OR 'patient* identification' OR 'patient* screening' OR 'patient* triage' OR 'patient* management' OR 'referral process' OR 'treatment pattern'/exp OR 'treatment algorithm'/exp OR 'apheresis' OR 'bridging therapy'/exp OR 'post$infusion' | 223287 |
| 46 | #43 OR #44 OR #45 | 1380104 |
| 47 | #23 OR #42 OR #46 | 6854594 |
| 48 | #4 AND #8 AND #47 | 1420 |
| 49 | #5 AND #47 AND australia:ad,ti,ab,ff | 253 |
| 50 | #48 OR #49 | 1618 |
| 51 | #50 AND [conference abstract]/lim AND [2020-2023]/py | 687 |
| 52 | #50 AND ([article]/lim OR [article in press]/lim) | 364 |
| 53 | #51 OR #52 | 1051 |

Table S2. Search terms for MEDLINE and MEDLINE-IN-PROCESS via [www.pubmed.ncbi.nlm.nih.gov/](http://www.pubmed.ncbi.nlm.nih.gov/) (date of the search: March 28, 2023)

| No. | Query | Results |
| --- | --- | --- |
| 1 | (multiple myeloma[MeSH Terms]) OR ("multiple myeloma*") OR (myeloma*) OR (multiple-myeloma*) OR (multiple myeloma*[Title/Abstract]) OR ("multiple myeloma"[Title/Abstract:~1]) | 75,156 |
| 2 | (myeloma* plasma-cell) OR "plasma-cell myeloma*" OR "plasma cell myeloma" OR (cell myeloma* plasma) OR (myeloma* plasma cell) OR "plasma cell myeloma*" OR "myeloma-multiple" OR (myeloma* multiple*) | 61,700 |
| 3 | "kahler disease"[Title/Abstract:~1] OR myelomatosis | 61,037 |
| 4 | #1 OR #2 OR #3 | 75,169 |
| 5 | "antigen receptor* chimeric"[Title/Abstract:~1] OR "chimeric antigen receptor*" OR (chimeric AND (immunoreceptor* OR (t-cell receptor*) OR (t-cell receptor*))) OR "artificial t cell receptor*" OR "artificial t-cell receptor*" OR "receptor* chimeric t-cell*" OR car-t OR car-t cell* | 14,722 |
| 6 | "bb 2121" OR "ciltacabtagene autoleucel" OR "idecabtagene vicleucel" | 91 |
| 7 | "abecma" OR "jnj 68284528" OR "lcar-b38m car-t cells" OR "carvykti" OR "anti-bcma car t cell" OR "ide-cel" | 85 |
| 8 | #5 OR #6 OR #7 | 14,732 |
| 9 | clinical trial[MeSH Terms] | 381,133 |
| 10 | clinical trials, randomized[MeSH Terms] | 164,967 |
| 11 | controlled clinical trial[MeSH Terms] | 214,552 |
| 12 | multicenter trial[MeSH Terms] | 22,219 |
| 13 | ((clinical trial, phase i[MeSH Terms]) OR (clinical trial, phase ii[MeSH Terms])) OR (clinical trial, phase iii[MeSH Terms]) | 20,561 |
| 14 | randomization[MeSH Terms] | 106,913 |
| 15 | (method, single blind[MeSH Terms]) OR (method, double blind[MeSH Terms]) | 206,185 |
| 16 | crossover design[MeSH Terms] | 54,842 |
| 17 | "placebo" | 244,969 |
| 18 | ("randomised controlled trial*"[Title/Abstract] OR "randomized controlled trial*"[Title/Abstract]) | 246,535 |
| 19 | rct[Title/Abstract] OR "non rct"[Title/Abstract] OR "non-rct"[Title/Abstract] | 33,489 |
| 20 | placebo*[Title/Abstract] | 245,514 |
| 21 | "triple blinded"[Title/Abstract:~1] OR "triple blind"[Title/Abstract:~1] OR "single blind"[Title/Abstract:~1] OR "single blinded"[Title/Abstract:~1] OR "double blind"[Title/Abstract:~1] OR "double blinded"[Title/Abstract:~1] | 191,774 |
| 22 | #9 OR #10 OR #11 OR #12 OR #13 OR #14 OR #15 OR #16 OR #17 OR #18 OR #19 OR #20 OR #21 | 974,581 |
| 23 | "cross sectional study"[Title/Abstract:~1] OR "cross sectional studies"[Title/Abstract:~1] | 288,351 |
| 24 | "epidemiologic* study"[Title/Abstract:~1] OR "epidemiologic* studies"[Title/Abstract:~1] | 30,194 |
| 25 | "observational study"[Title/Abstract:~1] OR "observational studies"[Title/Abstract:~1] | 188,309 |
| 26 | "follow up study"[Title/Abstract:~1] OR "follow up studies"[Title/Abstract:~1] | 69,720 |
| 27 | "case control study"[Title/Abstract:~1] OR "case control studies"[Title/Abstract:~1] | 132,105 |
| 28 | "cohort study"[Title/Abstract:~1] OR "cohort studies"[Title/Abstract:~1] | 334,746 |
| 29 | analysis, cohort[MeSH Terms] | 2,461,062 |
| 30 | "prospective study" | 160,124 |
| 31 | "retrospective study" | 204,132 |
| 32 | longitudinal study[MeSH Terms] | 163,898 |
| 33 | "family study" | 4,787 |
| 34 | case control study[MeSH Terms] | 1,401,171 |
| 35 | "clinical study" | 68,647 |
| 36 | ((population register[MeSH Terms])) OR (registr*[Title/Abstract]) | 388,578 |
| 37 | "real world"[Title/Abstract:~3] | 74,926 |
| 38 | ("real world evidence"[Title/Abstract:~2]) OR ("real world research"[Title/Abstract:~2]) OR ("real world study"[Title/Abstract:~2]) OR ("real world studies"[Title/Abstract:~2]) OR ("real world trial"[Title/Abstract:~2]) OR ("real world trials"[Title/Abstract:~2]) | 14,003 |
| 39 | "hospital record"[Title/Abstract:~3] OR "hospital records"[Title/Abstract:~3] | 20,300 |
| 40 | "claim medical"[Title/Abstract:~3] OR "claim database"[Title/Abstract:~3] OR "claim databases"[Title/Abstract:~3] OR "claims medical"[Title/Abstract:~3] | 5,180 |
| 41 | #23 OR #24 OR #25 OR #26 OR #27 OR #28 OR #29 OR #30 OR #31 OR #32 OR #33 OR #34 OR #35 OR #36 OR #37 OR #38 OR #39 OR #40 | 3,641,427 |
| 42 | "guideline*" OR "practice guideline*" OR "clinical decision rule*" OR (clinical pathway[MeSH Terms]) OR (clinical protocol[MeSH Terms]) OR (consensus[MeSH Terms]) | 800,700 |
| 43 | "position statement*"[Title/Abstract] OR "practice parameter*"[Title/Abstract] OR "policy statement*"[Title/Abstract] OR "best practice*"[Title/Abstract] OR "practice guideline"[Title/Abstract:~2] OR "practice guidelines"[Title/Abstract:~2] OR "treatment guideline"[Title/Abstract:~2] OR "treatment guidelines"[Title/Abstract:~2] OR "treatments guideline"[Title/Abstract:~2] OR "treatments guidelines"[Title/Abstract:~2] OR "clinical guideline"[Title/Abstract:~2] OR "clinical guidelines"[Title/Abstract:~2] | 128,964 |
| 44 | "patient* selection" OR "patient* identification" OR "patient* screening" OR "patient* triage" OR "patient* management" OR "referral process" OR "treatment pattern" OR "treatment algorithm" OR "apheresis" OR "bridging therapy" OR "post infusion" OR "post-infusion" | 139,599 |
| 45 | #42 OR #43 OR #44 | 951,374 |
| 46 | #22 OR #41 OR #45 | 5,064,820 |
| 47 | #4 AND #8 AND #46 | 321 |
| 48 | (#5 AND #46 ) AND (Australia) | 52 |
| 49 | #47 OR #48 | 365 |

Table S3. PICOS Framework and eligibility criteria for the SLR

| Category | Inclusion criteria | Exclusion criteria |
| --- | --- | --- |
| Population | Patients with MM treated with CAR-T cell therapy, including all types of subgroups such as age groups, different types of myeloma, refractory/relapsed MM, etc.  Adult populations outside of MM who have received CAR-T cell therapy only if the studies have authors with Australian affiliations | Populations not listed for inclusion |
| Interventions | CAR-T cell therapy (Idecabtagene Vicleucel and Ciltacabtagene Autoleucel) for all studies  For studies of other CAR-T cell treatments, only studies with authors with affiliation to Australian institutions | Interventions other than the CAR-T therapies listed for inclusion |
| Comparator | No restrictions | No restrictions |
| Outcomes | Outcomes of interest include (not exhaustive):  Diagnosis:   - Screening recommendations and practices - Triage methods - Identification of patients   Patient management:   - Referral process - Timing of apheresis - Treatment patterns and algorithms (including therapies used before and after CAR-T cell therapy) - Post-infusion management of CAR-T cell therapy - Percentage of patients receiving CAR-T as “off-label” treatment (including out of specification) - Management of CAR-T patients post-infusion as outpatients (i.e., not admitted to the hospital) | Other outcomes of interest not listed |
| Study type | Observational/real world evidence studies, interventional studies, guidelines, expert consensus, advisory board publications | Case report, case series |
| Publication type | Peer-reviewed journal article, conference abstract (2020 onward), report, guideline, review | Editorials, note, news article, letters |
| Language | No restrictions | Not applicable |
| Geographical scope | No restrictions | Not applicable |
| Publication year | No restrictions | Not applicable |

Abbreviations: CAR-T, chimeric antigen receptor T-cell; MM, multiple myeloma.

# Included Studies from SLR

Table S4. Summary of included studies

| Author (year) | Study type | Region | CAR-T cell therapy | Objectives | Eligibility criteria |
| --- | --- | --- | --- | --- | --- |
| Mi (2023) (3) | Phase II clinical trial  CARTIFAN-1 (NCT03758417) | China | Cilta-cel | To evaluate the efficacy and safety of cilta-cel in Chinese patients with RRMM | Age ≥18 years  RRMM  IMWG criteria  ≥3 previous LOT  ECOG PS 0 or 1 |
| Cohen (2023) (4) | Phase II clinical trial  CARTITUDE-2 (NCT04133636) | USA, Europe, Israel and Saudi Arabia | Cilta-cel | To evaluate the overall minimal residual disease negative rate of participants with progressive MM who receive cilta-cel | Age ≥18 years  RRMM  IMWG criteria  ECOG PS 0 or 1 |
| Cohen (2022) (5) | Phase Ib/II clinical trial  CARTITUDE-1 (NCT03548207) | USA, Japan | Cilta-cel | To assess the associated factors for neurocognitive treatment-emergent adverse events | Age ≥18 years  RRMM  IMWG criteria  ≥3 previous LOT  ECOG PS 0 or 1 |
| Wong (2021) (6) | Phase Ib/II clinical trial  CARTITUDE-1 (NCT03548207) | USA and Japan | Cilta-cel | To report the institutional experiences of anakinra use in the management of CRS in patients who have received cilta-cel | Age ≥18 years  RRMM  IMWG criteria  ≥3 previous LOT  ECOG PS 0 or 1 |
| Einsele (2021) (7) | Phase II clinical trial  CARTITUDE-2 (NCT04133636) | USA, Europe, Israel, and Saudi Arabia | Cilta-cel | To describe the mitigation and management strategies implemented to identify and reduce the risk for neurologic adverse events in Cohort A (progressive MM after 1−3 prior lines of therapy) | Age ≥18 years  MM  IMWG criteria  1 to 3 previous LOT  ECOG PS 0 or 1 |
| Rodriguez-Otero (2023) (8) | Phase III clinical trial  KarMMa-3 (NCT03651128) | USA, Canada, Japan, and Europe | Ide-cel | To evaluate the CAR-T cell therapy ide-cel as compared with standard regimens in patients with triple-class–exposed Refractory MM | Age ≥18 years  Refractory MM  2 to 4 previous LOT  ECOG PS 0 or 1 |
| Munshi (2021) (9) | Phase II clinical trial  KarMMa (NCT03361748) | USA, Canada, Japan, and Europe | Ide-cel | To confirm the efficacy and safety of ide-cel in patients with relapsed and refractory myeloma | Age ≥18 years  RRMM  IMWG criteria  ≥3 previous LOT  ECOG PS 0 or 1 |
| Rodriguez-Otero (2021) (10) | Phase II clinical trial  KarMMa (NCT03361748) | USA, Canada, Japan, and Europe | Ide-cel | To evaluate subsequent anti-myeloma therapy after ide-cel, treatment in patients with RRMM | Age ≥18 years  Refractory MM  2 to 4 previous LOT  ECOG PS 0 or 1 |
| Hansen (2023) (11) | Real-world experience | USA | Ide-cel | To report clinical outcomes with ide-cel under the commercial Food and Drug Administration label | RRMM  ≥4 previous LOT |
| Logue (2022) (12) | Multicenter retrospective study | USA | Ide-cel | To characterize early cellular and humoral reconstitution, infections, and need for supportive therapies due to treatment-related cytopenias in patients with RRMM who received ide-cel in the commercial setting | RRMM |
| Hansen (2022) (13) | Real-world experience | USA | Ide-cel | To evaluate the real-world outcomes of patients treated with ide-cel under the commercial FDA label | Age ≥18 years  RRMM  IMWG criteria  ≥3 previous LOT  ECOG PS 0 or 1 |
| Canonico (2022) (14) | Real-world experience | USA | Ide-cel | To report our initial experience with commercial ide-cel use at the Dana-Farber Cancer Institute/Brigham and Women’s Hospital | NR |
| Akhoundova (2022) (15) | Real-world experience | Switzerland | Ide-cel | To report the first real-world experiences in MM patients treated with commercial ide-cel | RRMM |
| Kourelis (2023) (16) | Real-world experience | USA | Ide-cel and Cilta-cel | To evaluate the extent of limited access to CAR-T cell therapy in real world and how centers are handling the challenges of CAR-T slot allocation in the US | MM CAR-T experts |
| Banerjee (2021) (17) | Single center retrospective study | USA | Idecabtagene vicleucel, bb21217, ciltacabtagene autoleucel, or orvacabtagene autoleucel | To identify whether shorter time-to-tocilizumab intervals have any impact on therapy-related toxicities or clinical outcomes among patients with RRMM receiving CAR-T therapies | Age ≥18 years  MM  ≥3 previous LOT |
| Giri (2021) (18) | Real-world experience | USA | Non-specific | To evaluate real-world applicability of commercial chimeric CAR-T cell therapy among older adults with RRMM | Newly diagnosed MM  ≥1 previous LOT |
| Carty (2022) (20) | Real-world experience | Australia | Non-specific | To assess the nutritional status of patients before CAR-T cell therapy and observe post-treatment nutritional status, symptoms and dietetic interventions | NR |
| Perram (2022) (21) | Position statement | Australia and New Zealand | NA | COVID-19 management in patients with haemopoietic stem cell transplant and CAR-T | NA |
| Lapidus (2022) (22) | Systematic literature review | NA | NA | Neuroimaging findings in immune effector cell associated neurotoxicity syndrome after chimeric antigen receptor T-cell therapy | NA |
| Tam (2022) (23) | Letter to the editor | Australia | NA | Fitness criteria for Australian patients referred for CAR-T-cell therapy | NA |
| Dowling (2022) (24) | Commentary | NA | NA | Post CAR-T cytopenia: poorly understood and clinically challenging | NA |

Abbreviations: CAR-T, chimeric antigen receptor T; cilta-cel, ciltacabtagene autoleucel; COVID-19, coronavirus disease 2019; FDA, Food and Drug Administration; ide-cel, idecabtagene vicleucel; IMWG, International Myeloma Working Group; LOT, lines of therapy; MM, multiple myeloma; RRMM, relapsing remitting multiple myeloma

# Detailed results of Delphi panel survey

Consensus was defined as 70% conformity of panelist responses. Panelists were asked to agree or disagree whether a statement should be considered by practitioners involved in CAR-T cell therapy. Statements that did not reach consensus in the first survey were included in the second round. In total, seven panelists completed the first survey, and eight panelists completed the second survey.

Table S5. Summary of Delphi survey responses on patient eligibility criteria for CAR-T cell therapy

| **Factor** | **Agreement** | | **Importance** | | | **Practicality** | | |
| --- | --- | --- | --- | --- | --- | --- | --- | --- |
|  | **Proportion of panelists** | **Consensus decision** | **Very Important** | **Somewhat important** | **Not important** | **Very practical** | **Somewhat practical** | **Not practical** |
| **Patient-related factors:** | | | | | | | | |
| Adequate cardiac function | 100% | Agree, consensus reached | 50.0% | 50.0% | 0 | 75.0% | 25.0% | 0 |
| Adequate respiratory function | 85.7% | Agree, consensus reached | 25.0% | 75.0% | 0 | 75.0% | 12.5% | 12.5% |
| Adequate renal function | 71.4% | Agree, consensus reached | 25.0% | 62.5% | 0 | 87.5% | 0 | 12.5% |
| Adequate liver function | 71.4% | Agree, consensus reached | 37.5% | 50.0% | 12.5% | 87.5% | 12.5% | 0 |
| Life expectancy | 85.7% | Agree, consensus reached | 25.0% | 75.0% | 0 | 50.0% | 50.1% | 0 |
| Frailty score | 71.4% | Agree, consensus reached | 37.5% | 62.5% | 0 | 62.5% | 12.5% | 25% |
| Absence of active, uncontrolled infection | 100% | Agree, consensus reached | 37.5% | 50.0% | 12.6% | 50.0% | 50.0% | 0 |
| Available social support | 87.5% | Agree, consensus reached | 28.3% | 57.1% | 14.3% | 14.3% | 57.1% | 28.6% |
| Baseline cognitive status | 62.5% | No consensus | NA | NA | NA | NA | NA | NA |
| Age | 25.0% | Disagree, consensus reached | NA | NA | NA | NA | NA | NA |
| **Disease-related factors:** | | | | | | | | |
| ECOG score | 100% | Agree, consensus reached | 62.5% | 37.5% | 0 | 87.5% | 12.5% | 0 |
| Meet criteria of RRMM | 71.4% | Agree, consensus reached | 87.5% | 0 | 12.5% | 87.5% | 0 | 12.5% |
| Pace of disease progression | 85.7% | Agree, consensus reached | 75.0% | 25.0% | 0 | 50.0% | 50.0% | 0 |
| Prior exposed lines of treatment | 71.4% | Agree, consensus reached | 12.5% | 37.5% | 50.0% | 62.5% | 12.5% | 25.0% |
| Absence of active, uncontrolled CNS diseases | 87.5% | Agree, consensus reached | 50.0% | 40.0% | 20.0% | 42.9% | 42.9% | 14.3% |
| Availability of effective bridging therapy if this is required | 62.5% | No consensus | NA | NA | NA | NA | NA | NA |
| Prior refractory line of treatment | 12.5% | Disagree, consensus reached | NA | NA | NA | NA | NA | NA |
| Most current clinical response during referral | 14.3% | Disagree, consensus reached | NA | NA | NA | NA | NA | NA |
| Absence of markers of high-risk cytogenetics | 14.3% | Disagree, consensus reached | NA | NA | NA | NA | NA | NA |
| Absence of extra-medullary disease | 14.3% | Disagree, consensus reached | NA | NA | NA | NA | NA | NA |

Abbreviations: CMV, cytomegalovirus; CNS, central nervous system; EBV, Epsteine-Barr virus; ECOG, Eastern Cooperative Oncology Group; HBV, hepatitis B virus; HCV, hepatitis C virus; HIV, human immunodeficiency virus; LVEF, Left ventricular ejection fraction; MSAC, Medical and Scientific Advisory Council; NA, not applicable; RRMM, refractory-relapsing multiple myeloma

Table S6. Summary of Delphi survey responses on patient exclusion criteria for CAR-T cell therapy

| **Factor** | **Agreement** | | **Importance** | | |
| --- | --- | --- | --- | --- | --- |
|  | **Proportion of panelists** | **Consensus decision** | **Very Important** | **Somewhat important** | **Not important** |
| Active or poorly controlled CNS disorder (including epilepsy, dementia, or CNS involved autoimmune disorder) | 85.7% | Agree, consensus reached | 62.5% | 37.5% | 0 |
| Active infection with hepatitis virus (HBV, HCV) and/or others (e.g., EBV, CMV) | 85.7% | Agree, consensus reached | 100% | 0 | 0 |
| Human immunodeficiency virus (HIV) | 71.4% | Agree, consensus reached | 75% | 25% | 0 |
| Other active infection (bacterial, fungal, etc.) | 71.4% | Agree, consensus reached | 87.5% | 12.5% | 0 |
| Live vaccines within 6 weeks of planned CAR-T cell infusion | 71.4% | Agree, consensus reached | 50% | 50% | 0 |
| Comorbidities conferring an expected life expectancy of <5 years (e.g. secondary malignancies) | 71.4% | Agree, consensus reached | 50% | 50% | 0 |
| Active, uncontrolled graft-versus-host disease | 71.4% | Agree, consensus reached | 62.5% | 25% | 12.5% |
| Complex psychological issues that may impact compliance or safety | 100% | Agree, consensus reached | 37.5% | 50% | 12.5% |
| Prior BCMA therapy | 0% | Disagree, consensus reached | NA | NA | NA |
| Renal dysfunction | 28.6% | Disagree, consensus reached | NA | NA | NA |
| Prior treatment with allo-HSCT | 28.6% | Disagree, consensus reached | NA | NA | NA |
| Cardiovascular disease | 28.6% | Disagree, consensus reached | NA | NA | NA |

Abbreviations: BCMA, B-cell maturation antigen; CAR-T, chimeric antigen receptor T-cell; CMV, cytomegalovirus; CNS, central nervous system; EBV, Epstein-Barr virus; HBV, hepatitis B virus; HCV, hepatitis C virus; HIV, human immunodeficiency virus; NA, not applicable; TB, tuberculosis

Table S7. Summary of Delphi survey responses on patient prioritization for CAR-T cell therapy

| **Factor** | **Agreement** | | **Importance** | | | **Practicality** | | |
| --- | --- | --- | --- | --- | --- | --- | --- | --- |
|  | **Proportion of panelists** | **Consensus decision** | **Very Important** | **Somewhat important** | **Not important** | **Very practical** | **Somewhat practical** | **Not practical** |
| **Patient-related factors:** | | | | | | | | |
| Disease burden | 85.7% | Agree, consensus reached | 50% | 25% | 25% | 75% | 25% | 0 |
| Disease aggressiveness | 85.7% | Agree, consensus reached | 50% | 25% | 25% | 37.5% | 50% | 12.5% |
| Bridgeability | 100% | Agree, consensus reached | 75.0% | 25.0% | 0 | 50.0% | 50.0% | 0 |
| Absence of active, uncontrolled infection | 87.5% | Agree, consensus reached | 87.5% | 12.5% | 0 | 75.0% | 25.0% | 0 |
| Age | 25.0% | Disagree, consensus reached | NA | NA | NA | NA | NA | NA |
| Time spent on the waiting list | 14.3% | Disagree, consensus reached | NA | NA | NA | NA | NA | NA |
| Hematopoietic cell transplantation | 14.3% | Disagree, consensus reached | NA | NA | NA | NA | NA | NA |
| **Disease-related factors:** | | | | | | | | |
| Availability of alternative treatment options | 71.4% | Agree, consensus reached | 0 | 100% | 0 | 66.7% | 16.67% | 16.7% |
| Access to appropriate social support (e.g. caregiver) | 100% | Agree, consensus reached | 62.5% | 37.5% | 0 | 25.0% | 62.5% | 12.5% |
| System/regional capacity | 50.0% | No consensus | NA | NA | NA | NA | NA | NA |
| Geographical location limitations | 28.5% | Disagree, consensus reached | NA | NA | NA | NA | NA | NA |
| Equity and equality considerations | 14.3% | Disagree, consensus reached | NA | NA | NA | NA | NA | NA |

Abbreviation: NA, not applicable

Table S8. Summary of Delphi survey responses on panelist-suggested cut-off values and assessment timepoints

| **Factor** | **Measurement and cut-off values** | | **Timepoint of assessment** | |
| --- | --- | --- | --- | --- |
|  | **Panelist suggested strategy (proportion of panelists)** | **Consensus decision** | **Panelist suggested strategy (proportion of panelists)** | **Consensus decision** |
| Adequate cardiac function | LVEF (100%): ≥40% (87.5%); ≥50% (12.5%)  NYHA grade <2 + LVEF ≥40% (12.5%) | LVEF ≥40% | Pre-apheresis/pre-collection (100%)  At screening (37.5%)  Pre- lymphodepletion (25.0%) | Pre-apheresis |
| Adequate respiratory function | Oxygen saturation >90% (62.5%) | No consensus | At screening (75.0%)  Pre-apheresis/pre-collection (37.5%)  Pre-lymphodepletion (12.5%)  Post-lymphodepletion (12.5%)  At infusion (12.5%) | At time of screening |
| Adequate renal function | Creatinine clearance (87.5%): ≥30ml/min (75.0%); ≥40ml/min (25.0%)  eGFR≥30 (12.5%) | Creatinine clearance ≥30ml/min | At screening (50.0%)  Pre-apheresis/pre-collection (37.5%)  Pre-lymphodepletion (50.0%)  At lymphodepletion (37.5%)  Post-lymphodepletion (12.5%)  At infusion (12.5%) | No consensus |
| Adequate liver function | ALT/AST + total bilirubin (75.0%):  ALT/AST <5 x ULN, total bilirubin <2 x ULN (62.5%)  ALT/AST <3 x ULN, total bilirubin <3 x ULN (12.5%) | Consensus on use of ALT/AST + total bilirubin measurement, but not the cut-off value | Pre-apheresis/pre-collection (62.5%)  Pre-lymphodepletion (37.5%)  Post lymphodepletion (12.5%)  At infusion (12.5%) | No consensus |
| Expected life expectancy | >5 years (50.0%)  >6 months (12.5%)  >3 months (37.5%) | No consensus | Pre-apheresis/pre-collection (87.5%)  At infusion (25.0%) | Pre-apheresis |
| Frailty score | ECOG performance status 0-1 (62.5%)  IMWG Frailty Index as frail (37.5%)  Simplified Frailty Score≥2 (12.5%) | No consensus | Pre-apheresis/pre-collection (62.5%)  Pre-lymphodepletion (37.5%)  At infusion (37.5%) | No consensus |
| Absence of active, uncontrolled infection | HIV, HBV, HCV, CMV, EBV, syphilis, bacterial infections (100%)  As per TGA (12.5%)  QuantiFERON gold (50.0%) | Detection of HIV, HBV, HCV, CMV, EBV, syphilis, bacterial infections | Pre-apheresis/pre-collection (100%)  At lymphodepletion (75.0%)  At infusion (62.5%) | Pre-apheresis and at lymphodepletion |
| ECOG score | ECOG <2 (100%) | ECOG <2 | Pre-lymphodepletion (75.0%)  At infusion (25.0%) | Pre-lymphodepletion |
| Meet criteria of RRMM | As per IMWG criteria (62.5%)  As per MSAC wording (37.5%) | No consensus | NA | NA |
| Prior exposed lines of treatment | 3 classes (25.0%)  As per reimbursement criteria/MSAC wording (87.5%) | As per reimbursement criteria/MSAC wording | NA | NA |
| Absence of active CNS diseases | MRI and LP (12.5%) | No consensus | NA | NA |
| Full Blood count | Hemoglobin: nil (75.0%)  Platelets >50×10^9^/L (37.5%)  Lymphocytes (75.0%): >0.5×10^9^/L (50.0%); nil (25.0%) | Consensus on quantification of hemoglobin (nil cut-off value) and lymphocytes (no consensus cut-off value) | At referral (62.5%)  At infusion (50.0%)  Assess lymphocytes pre-apheresis and platelets pre-infusion (37.5%) | No consensus |
| Full biochemistry | Corrected serum calcium (75.0%): <3 mmol/L (12.5%); ULN (62.5%) | Consensus on quantification of corrected serum calcium, but not a cut-off value | NA | NA |
| Pregnancy test (if patients of childbearing age) | Negative (100%) | Negative | At infusion (50.0%)  Pre-apheresis/pre-collection (12.5%)  Monthly during treatment (37.5%) | No consensus |

Abbreviations: ALT, alanine aminotransferase; AST, aspartate aminotransferase; CMV, cytomegalovirus; CNS, central nervous system; ECOG, Eastern Cooperative Oncology Group; EBV, Epstein-Barr virus; HBV, hepatitis B virus, HCV, hepatitis C virus; HIV, human immunodeficiency virus; IMWG, International Myeloma Working Group; LP, lumbar puncture; LVEF, left ventricular ejection fraction; MSAC, Medicare Services Advisory Committee; MRI, magnetic resonance imaging; NA, not applicable; NYHA, New York Heart Association; RRMM, relapsed/refractory multiple myeloma

Table S9. Summary of Delphi survey responses on wash-out practices prior to leukapheresis

| **Factor** | **Agreement** | | **Importance** | | | **Minimum time period** | |
| --- | --- | --- | --- | --- | --- | --- | --- |
|  | **Proportion of panelists** | **Consensus decision** | **Very Important** | **Somewhat important** | **Not important** | **Panelist-suggested strategy (proportion of panelists)** | **Consensus decision** |
| Allo-HSCT (off immunosuppression and GvHD free) | 100% | Agree, consensus reached | 37.5% | 50.0% | 12.5% | 24 weeks (25.0%)  12 weeks (37.5%)  8 weeks (25%) | No consensus |
| Donor lymphocyte infusion | 71.4% | Agree, consensus reached | 50.0% | 50.0% | 0 | 8 weeks (75.0%)  4 weeks (25.0%) | 8 weeks |
| High-dose chemotherapy | 85.7% | Agree, consensus reached | 12.5% | 87.5% | 0 | 8 weeks (37.5%)  4 weeks (62.5%) | No consensus |
| Systemic corticosteroids | 85.7% | Agree, consensus reached | 50.0% | 50.0% | 0 | 2 weeks (50.0%)  3 to 7 days (50.0%) | No consensus |
| Proteasome inhibitors | 85.7% | Agree, consensus reached | 0 | 87.5% | 12.5% | 2 weeks (50.0%)  7 days (37.5%) | No consensus |
| Immunomodulatory drugs | 85.7% | Agree, consensus reached | 12.5% | 75.0% | 12.5% | 2 weeks (37.5%)  7 days (37.5%) | No consensus |
| Anti-CD38 monoclonal antibodies | 71.4% | Agree, consensus reached | 12.5% | 75.0% | 12.5% | 4 weeks (50.0%)  2 weeks (25.0%) | No consensus |
| Bendamustine | 87.5% | Agree, consensus reached | 75.0% | 12.5% | 12.5% | 12 weeks (37.5%)  24 weeks (50.0%) | No consensus |
| Radiotherapy | 37.5% | No consensus | NA | NA | NA | NA | NA |

Abbreviations: HSCT, hematopoietic stem cell transplantation; GvHD, graft-versus-host disease; NA, not applicable

Table S10. Summary of Delphi survey responses on factors to consider when deciding the type and duration of bridging therapy

| **Factor** | **Agreement** | | **Importance** | | | **Practicality** | | |
| --- | --- | --- | --- | --- | --- | --- | --- | --- |
|  | **Proportion of panelists** | **Consensus decision** | **Very Important** | **Somewhat important** | **Not important** | **Very practical** | **Somewhat practical** | **Not practical** |
| Disease aggressiveness | 85.7% | Agree, consensus reached | 100% | 0 | 0 | 62.5% | 37.5% | 0 |
| Disease burden | 85.7% | Agree, consensus reached | 62.5% | 37.5% | 0 | 62.5% | 37.5% | 0 |
| Historical CAR-T manufacturing time | 71.4% | Agree, consensus reached | 75.0% | 25.0% | 0 | 50.0% | 37.5% | 12.5% |
| Response to prior lines of therapy | 71.4% | Agree, consensus reached | 37.5% | 62.5% | 0 | 62.5% | 25.0% | 12.5% |
| Patient’s likelihood to tolerate bridging therapy | 71.4% | Agree, consensus reached | 87.5% | 12.5% | 0 | 50.0% | 50.0% | 0 |
| Availability of the intervention | 87.5% | Agree, consensus reached | 62.5% | 37.5% | 0 | 75.0% | 25.0% | 0 |
| Bridgeability | 87.5% | Agree, consensus reached | 87.5% | 25.0% | 0 | 62.5% | 37.5% | 0 |
| Prior lines of therapy used | 62.5% | No consensus | NA | NA | NA | NA | NA | NA |
| Age | 37.5% | No consensus | NA | NA | NA | NA | NA | NA |

Abbreviations: CAR-T, chimeric antigen receptor T-cell; NA, not applicable

Table S11. Summary of Delphi survey responses on statements related to lymphodepletion and infusion

| **Statement** | **Proportion of panelists** | **Consensus decision** |
| --- | --- | --- |
| **Lymphodepletion** | | |
| Lymphodeplete patients with the combination fludarabine + cyclophosphamide for 3 days | 85.7% | Agree, consensus reached |
| General doses for lymphodepletion are 25–30 mg/m^2^ for fludarabine and 250–300 mg/m^2^ for cyclophosphamide | 85.7% | Agree, consensus reached |
| Dose adjustment for fludarabine based on creatinine clearance | 71.4% | Agree, consensus reached |
| Consultation with a neurologist for patients with experience or risk of ICANS is the optimal practice if resources are available to do so | 62.5% | No consensus |
| **CAR-T cell infusion** | | |
| The use of paracetamol and antihistamine products to minimize infusion reactions is appropriate | 71.4% | Agree, consensus reached |
| CAR-T products that are out of specification can be used | 71.4% | Agree, consensus reached |
| It is necessary to wait for 48h after lymphodepletion before infusion | 100% | Agree, consensus reached |
| It is necessary to avoid using corticosteroids during infusion | 12.5% | Disagree, consensus reached |
| Hospitalization post CAR-T is a must | 0 | Disagree, consensus reached |

Abbreviations: CAR-T, chimeric antigen receptor T-cell; ICANS, immune effector cell-associated neurotoxicity syndrome

Table S12. Summary of Delphi responses on factors associated with movement and neurocognitive treatment emergent adverse event risk

| **Factor** | **Proportion of panelists** | **Consensus decision** |
| --- | --- | --- |
| Assessment of tumor burden at baseline | 71.4% | Agree, consensus reached |
| Presence of grade 2 or higher CRS | 75.0% | Agree, consensus reached |
| Assessment of CAR-T expansion and persistence | 50.0% | No consensus |
| Neurocognitive assessment before and after CAR-T infusion | 50.0% | No consensus |
| Assessment of absolute lymphocyte count | 100% | Agree, consensus reached |

Abbreviations: CAR-T, chimeric antigen receptor T-cell; CRS, cytokine release syndrome; MNT, movement and neurocognitive treatment emergent adverse event

Table S13. Summary of Delphi survey responses on monitoring schemes for adverse events and disease progression

| **Test or parameter** | **Agreement** | | **Panelist-suggested strategy that reached consensus (proportion of panelists)** |
| --- | --- | --- | --- |
|  | **Proportion of panelists** | **Consensus decision** |  |
| **Within 3 months post CAR-T infusion** | | | |
| CRS | 100% | Agree, consensus reached | Daily during first 14 days when hospitalized, then twice per week post discharge for first month, then every 4 weeks until 3 months (100%) |
| Full blood count | 85.7% | Agree, consensus reached | Daily during first 14 days when hospitalized, then twice per week post discharge for first month, then every 4 weeks until 3 months (87.5%)  Every visit for outpatient, or daily for inpatient (75%) |
| ICANS | 100% | Agree, consensus reached | No consensus |
| Infections | 100% | Agree, consensus reached | No consensus |
| MNTs, cognition | 75.0% | Agree, consensus reached | Daily during first 14 days when hospitalized, then twice per week post discharge for the first month, and then every 4 weeks for second and third 3 months (75.0%) |
| **From 3 months post CAR-T infusion** | | | |
| Full blood count | 71.4% | Agree, consensus reached | No consensus |
| Infections | 100% | Agree, consensus reached | No consensus |
| Neurological status | 100% | Agree, consensus reached | Every visit (75.0%) |
| MNTs, cognition | 75.0% | Agree, consensus reached | Every 3 months |
| Delayed tumor lysis syndrome/CRS/ICANS | 50.0% | No consensus | No consensus |
| MRI | 28.5% | Disagree, consensus reached | NA |
| **Clinical response** | | | |
| Serum free light chain | 100% | Agree, consensus reached | No consensus |
| Serum M protein quantification | 100% | Agree, consensus reached | No consensus |
| Standard follow-up | 85.7% | Agree, consensus reached | No consensus |
| Minimal residual disease status | 71.4% | Agree, consensus reached | No consensus |
| Serum immunofixation | 100% | Agree, consensus reached | No consensus |
| PET-CT | 75.0% | Agree, consensus reached | As clinically indicated (75.0%) |
| Bone marrow assessment | 87.5% | Agree, consensus reached | Upon indication, unexplained cytopenia, suspicion of secondary BM malignancies, or progressive disease (75.0%) |
| CAR-T persistence monitoring | 12.5% | Disagree, consensus reached | NA |
| MRI | 28.5% | Disagree, consensus reached | NA |

Abbreviations: CAR-T, chimeric antigen receptor T-cell; CRS, cytokine release syndrome; ICANS, immune effector cell-associated neurotoxicity syndrome; MoCA, Montreal Cognitive Assessment; MNT, movement and neurocognitive treatment emergent adverse event; MRI, magnetic resonance imaging; NA, not applicable; PET-CT, positron emission tomography–computed tomography

# References

1. McKenzie JE, Brennan SE, Ryan RE, Thomson HJ, Johnston RV, Thomas J. “Defining the criteria for including studies and how they will be grouped for the synthesis.,” In: Higgins JPT, Thomas J, Chandler J, Cumpston M, Li T, Page MJ, Welch VA, editors. *Cochrane Handbook for Systematic Reviews of Interventions*. Wiley (2019). p. 33–65 doi: 10.1002/9781119536604.ch3

2. Page MJ, McKenzie JE, Bossuyt PM, Boutron I, Hoffmann TC, Mulrow CD, Shamseer L, Tetzlaff JM, Akl EA, Brennan SE, et al. The PRISMA 2020 statement: an updated guideline for reporting systematic reviews. *Syst Rev* (2021) 10:89. doi: 10.1186/s13643-021-01626-4

3. Mi J-Q, Zhao W, Jing H, Fu W, Hu J, Chen L, Zhang Y, Yao D, Chen D, Schecter JM, et al. Phase II, open-label study of ciltacabtagene autoleucel, an anti–B-cell maturation antigen chimeric antigen receptor–T-cell therapy, in Chinese patients with relapsed/refractory multiple myeloma (CARTIFAN-1). *J Clin Oncol* (2023) 41:1275–1284. doi: 10.1200/JCO.22.00690

4. Cohen AD, Mateos M-V, Cohen YC, Rodriguez-Otero P, Paiva B, van de Donk NWCJ, Martin T, Suvannasankha A, De Braganca KC, Corsale C, et al. Efficacy and safety of cilta-cel in patients with progressive multiple myeloma after exposure to other BCMA-targeting agents. *Blood* (2023) 141:219–230. doi: 10.1182/blood.2022015526

5. Cohen AD, Parekh S, Santomasso BD, Gállego Pérez-Larraya J, van de Donk NWCJ, Arnulf B, Mateos M-V, Lendvai N, Jackson CC, De Braganca KC, et al. Incidence and management of CAR-T neurotoxicity in patients with multiple myeloma treated with ciltacabtagene autoleucel in CARTITUDE studies. *Blood Cancer J* (2022) 12:32. doi: 10.1038/s41408-022-00629-1

6. Wong SW, Richard S, Lin Y, Madduri D, Jackson CC, Zudaire E, Romanov V, Trigg M, Vogel M, Garrett A, et al. Anakinra targeting cytokine release syndrome associated with chimeric antigen receptor t-cell therapies. *Blood* (2021) 138:2812–2812. doi: 10.1182/blood-2021-150592

7. Einsele H, Parekh SS, Madduri D, Santomasso B, Gállego Pérez De Larraya J, van de Donk NWCJ, Arnulf B, Mateos M-V, De Braganca KC, Varsos H, et al. Incidence, mitigation, and management of neurologic adverse events in patients with multiple myeloma (MM) treated with ciltacabtagene autoleucel (cilta-cel) in CARTITUDE-2. *J Clin Oncol* (2021) 39:8028–8028. doi: 10.1200/JCO.2021.39.15_suppl.8028

8. Rodriguez-Otero P, Ailawadhi S, Arnulf B, Patel K, Cavo M, Nooka AK, Manier S, Callander N, Costa LJ, Vij R, et al. Ide-cel or standard regimens in relapsed and refractory multiple myeloma. *N Engl J Med* (2023) 388:1002–1014. doi: 10.1056/NEJMoa2213614

9. Munshi NC, Anderson LD, Shah N, Madduri D, Berdeja J, Lonial S, Raje N, Lin Y, Siegel D, Oriol A, et al. Idecabtagene vicleucel in relapsed and refractory multiple myeloma. *N Engl J Med* (2021) 384:705–716. doi: 10.1056/NEJMoa2024850

10. Rodriguez-Otero P, San-Miguel JF, Anderson LD Jr, Lonial S, Truppel-Hartmann A, Sanford J, Rowe E, Campbell TB, Munshi NC. Subsequent anti-myeloma therapy after idecabtagene vicleucel (ide-cel, bb2121) treatment in patients with relapsed/refractory multiple myeloma from the KarMMa study. *Blood* (2021) 138:2743–2743. doi: 10.1182/blood-2021-147990

11. Hansen DK, Sidana S, Peres LC, Colin Leitzinger C, Shune L, Shrewsbury A, Gonzalez R, Sborov DW, Wagner C, Dima D, et al. Idecabtagene vicleucel for relapsed/refractory multiple myeloma: real-world experience from the myeloma CAR T consortium. *J Clin Oncol* (2023) 41:2087–2097. doi: 10.1200/JCO.22.01365

12. Logue JM, Peres LC, Hashmi H, Colin-Leitzinger CM, Shrewsbury AM, Hosoya H, Gonzalez RM, Copponex C, Kottra KH, Hovanky V, et al. Early cytopenias and infections after standard of care idecabtagene vicleucel in relapsed or refractory multiple myeloma. *Blood Adv* (2022) 6:6109–6119. doi: 10.1182/bloodadvances.2022008320

13. Hansen DK, Sidana S, Peres L, Shune L, Sborov DW, Hashmi H, Kocoglu MH, Atrash S, Simmons G, Kalariya N, et al. Idecabtagene vicleucel (ide-cel) chimeric antigen receptor (CAR) T-cell therapy for relapsed/refractory multiple myeloma (RRMM): Real-world experience. *J Clin Oncol* (2022) 40:8042–8042. doi: 10.1200/JCO.2022.40.16_suppl.8042

14. Canonico D, Laubach J, Mo C, Sperling A, Nikiforow S, Redd R, Ramsdell L, McDermott K, Finn K, Desnoyers L, et al. P-006: Real world experience of patients treated with idecabtagene vicleucel: a BCMA-directed chimeric antigen receptor T-cell therapy for multiple myeloma. *Clin Lymphoma Myeloma Leuk* (2022) 22:S39. doi: 10.1016/S2152-2650(22)00336-6

15. Akhoundova Sanoyan D, Bacher U, Kronig M-N, Seipel K, Daskalakis M, Pabst T. Single-center experience with CAR T-cell therapy with idecabtagene vicleucel (ide-cel) for triple-class exposed relapsed/refractory multiple myeloma. *Swiss Med Wkly* (2022) 152 Suppl. 265:64 S. doi: https://doi.org/10.57187/smw.2022.40034

16. Kourelis T, Bansal R, Berdeja J, Siegel D, Patel K, Mailankody S, Htut M, Shah N, Wong SW, Sidana S, et al. Ethical Challenges with Multiple Myeloma BCMA Chimeric Antigen Receptor T Cell Slot Allocation: A Multi-Institution Experience. *Transplant Cell Ther* (2023) doi: 10.1016/j.jtct.2023.01.012

17. Banerjee R, Marsal J, Huang C-Y, Lo M, Kambhampati S, Kennedy VE, Arora S, Wolf JL, Martin TG, Wong SW, et al. Early time-to-tocilizumab after B cell maturation antigen-directed chimeric antigen receptor T cell therapy in myeloma. *Transplant Cell Ther* (2021) 27:477.e1-477.e7. doi: 10.1016/j.jtct.2021.03.004

18. Giri S, Bal S, Godby KN, Ravi G, Clark D, Ubersax C, Cooley A, White P, Rangarajan S, Williams GR, et al. Real-world applicability of commercial chimeric antigen receptor T cell therapy among older adults with relapsed and/or refractory multiple myeloma. *Blood* (2021) 138:4107–4107. doi: 10.1182/blood-2021-153796

19. Velickovic ZM, Rasko JEJ. Establishing a robust chimeric antigen receptor T-cell therapy program in Australia: the Royal Prince Alfred Hospital experience. *Cytotherapy* (2022) 24:45–48. doi: 10.1016/j.jcyt.2021.06.005

20. Carty D, Ismail H, Traer E, Riedel B, O’Leary N, Harrison S, Crowe J, Kotowicz E, Edbrooke L, Ftanou M, et al. Nutritional status of patients undergoing Chimeric Antigen Receptor T-Cell (CAR-T) therapy; a feasibility study. *Asia Pac J Clin Oncol* (2022) 18:174. doi: 10.1111/ajco.13869

21. Perram J, Purtill D, Bajel A, Butler J, O’Brien T, Teh B, Gilroy N, Ho PJ, Doocey R, Hills T, et al. Australia and New Zealand Transplant and Cellular Therapies (ANZTCT) position statement: COVID-19 management in patients with haemopoietic stem cell transplant and chimeric antigen receptor T cell. *Intern Med J* (2023) 53:119–125. doi: 10.1111/imj.15978

22. Lapidus AH, Anderson MA, Harrison SJ, Dickinson M, Kalincik T, Lasocki A. Neuroimaging findings in immune effector cell associated neurotoxicity syndrome after chimeric antigen receptor T-cell therapy. *Leuk Lymphoma* (2022) 63:2364–2374. doi: 10.1080/10428194.2022.2074990

23. Tam CS, Ho PJ, Purtill D, Blyth E, Butler J, Dickinson M, Harrison S. Fitness criteria for Australian patients referred for chimeric antigen receptor T-cell therapy. *Intern Med J* (2022) 52:1454–1456. doi: 10.1111/imj.15868

24. Dowling MR, Dickinson M. Post CAR-T cytopenia: poorly understood and clinically challenging. *Leuk Lymphoma* (2022) 63:1774–1776. doi: 10.1080/10428194.2022.2095631
